# Y-specific *amh* allele, *amhy*, is the master sex-determining gene in Japanese flounder *Paralichthys olivaceus*

**DOI:** 10.3389/fgene.2022.1007548

**Published:** 2022-09-16

**Authors:** Ricardo Shohei Hattori, Keiichiro Kumazawa, Masatoshi Nakamoto, Yuki Nakano, Toshiya Yamaguchi, Takeshi Kitano, Eiichi Yamamoto, Kanako Fuji, Takashi Sakamoto

**Affiliations:** ^1^ Department of Marine Biosciences, Tokyo University of Marine Science and Technology, Tokyo, Japan; ^2^ Nansei Field Station, National Research and Development Agency, Japan Fisheries Research and Education Agency, Mie, Japan; ^3^ Department of Biological Sciences, Graduate School of Science and Technology, Kumamoto University, Kumamoto, Japan; ^4^ Tottori Prefectural Fisheries Experimental Station, Tottori, Japan

**Keywords:** testis-determining gene, *amhy*, *amhx*, sex determination, müllerian-inhibiting substance

## Abstract

Japanese flounder (*Paralichthys olivaceus*) is an important marine fish species of both fisheries and aquaculture in Northeast Asia. The commercial interest for all-female progenies due to several sex-related traits has prompted basic research on the mechanisms of sex determination in this species. By conducting a linkage analysis of the sex-determining locus, we initially identified 12 microsatellite markers linked to sex in 11 scaffolds, whose localization was restricted to a specific region of linkage group 9. Sequence analysis of this region identified 181 genes based on the UniProt database annotations. Among them, the *amh* gene was considered a potential candidate for sex determination because this gene is known to have taken over the role of sex determination in many teleosts. An in-depth sequence analysis of both the coding and non-coding regions of *amh* in XX and XY individuals detected nine SNPs linked with maleness. However, because these substitutions were synonymous, the upstream and downstream regions of *amh* were also investigated and a male-specific variant with deletions in the promoter region was detected. This truncated Y-specific *amh* variant was named *amhy*, and the *amh* shared by both sexes was named *amhx*. The association analysis using both females and males of the genotypic sex inferred by the presence/absence of *amhy* found complete association with phenotypic sex and genotype. Gene expression analysis in larvae derived from a single-pair progeny by quantitative real-time PCR detected *amhy* transcripts in the larval trunks between 20 and 100 days after hatching only in XY larvae. Localization of *amhy* by *in situ* hybridization was detected in presumptive Sertoli cells of XY gonads. Expression of *amhx* was almost undetectable in both XX and XY genotypes. Loss of Amh function by CRISPR-Cas9 induced male-to-female sex reversal, indicating that this gene was necessary for the masculinization of XY individuals. In conclusion, the complete linkage of *amhy* with males, its early expression in XY gonads before testicular differentiation, and the induction of sex reversal by loss-of-function mutation support the view that *amhy* is the sex-determining gene in this species.

## Introduction

Sex differentiation of the gonads in gonochoristic species is a binary, antagonistic process determined by the balance between female-promoting and male-promoting factors, which culminates in the commitment of the undifferentiated gonad into either an ovary or testis (Hattori et al., 2020). In fish, this process is influenced by genotypic and environmental factors wherein the strength of one over another may differ greatly according to the species or taxonomic group (Fernandino et al., 2013).

Research on genotypic factors of sex determination in teleosts has unraveled a large repertoire of sex-determining genes, including the canonical transcription factors with DM- or SOX-binding domains as well as some unusual players such as the immune-related *sdY* of salmonids (Yano et al., 2013) and the steroidogenic enzyme *hsd17b1* in *Seriola quinqueradiata* (Koyama et al., 2019). Despite such variation, there is seemingly a high likelihood that members of the TGF-beta gene superfamily will be recruited as sex-determining genes, particularly those related to Amh-signaling. Y-specific *amh* duplication (namely *amhy*) has been reported in some Atheriniformes (Hattori et al., 2012; Yamamoto et al., 2014; Bej et al., 2017; Hattori et al., 2019), in Esociformes (Pan et al., 2019), and in the Cichliformes Nile tilapia (Li et al., 2015). In some fugu species (*Takifugu* sp.), *amhrII*, the receptor that is supposed to bind to *amh*, has been proposed as the key sex-determining gene (Ieda et al., 2018).

Despite the presence of well-known genotypic sex determinants of sex, in some species environmental cues can overcome the course of sex differentiation, inducing the appearance of male-to-female or female-to-male sex-reversed fish (Hattori et al., 2020). Currently, both (genotypic and environmental) sex determination systems are considered as extremes of a continuum rather than two independent and mutually exclusive mechanisms (Yamamoto et al., 2014) because they seem to coexist even in wild populations (Baroiller and D’Cotta, 2016; Miyoshi et al., 2020). The physiological and molecular mechanisms governing thermolabile sex determination have advanced remarkably since the discovery of the link between the stress hormone cortisol and masculinization in teleosts (Hattori et al., 2009; Hayashi et al., 2010; Yamaguchi et al., 2010). Cortisol is produced by the adrenal glands and its release is stimulated by Crh signaling (the ligand Crhb and the receptors Crhr1 and Crhr2) during masculinization of XX genotypes in Japanese medaka (Castañeda Cortés et al., 2019), suggesting that the central nervous system has an important role in gonadal sex determination induced by the environment.

Japanese flounder (*Paralichthys olivaceus*) is a marine flatfish with high commercial importance for fisheries and aquaculture in Northeast Asia (Japan, Korea, and China). Sex determination is controlled both genotypically by a XX-XY male heterogametic system (Yamamoto, 1999) and environmentally by sex steroids and water temperature (Hayashi et al., 2010). The latter can affect sex determination by driving undifferentiated gonad into either ovary or testis when exposure occurs before fish reach 40 mm of total body length (Yamamoto, 1999). According to this study, temperatures below 17.5°C or above 22.5°C were associated to high percentage of males. All XX progeny production by gynogenesis or crosses between XX genotypes (neomales with normal females) has been adopted to improve female production because females generally have better growth rates than males after sexual maturation; other female-specific traits such as lower susceptibility to diseases are also factors behind the preference for females in most fish species (Peterson and David, 2012). However, despite the absence of male genetic factors in these progeny, some individuals undergo natural female-to-male sex reversal, yielding undesired phenotypic males among the XX progenies (Hayashi et al., 2010). Warm temperature of approximately 22°C is likely the major factor that affects masculinization, but other stressors such as background color may also be involved, as was demonstrated in *Paralichthys lethostigma* (Mankiewicz et al., 2013).

Thus, basic information on genotypic sex determination is important for better understanding the genetic molecular mechanisms governing sex determination and for providing molecular tools for the development of reproductive biotechnologies in *P. olivaceus*. Considering that environmental sex determination is also strong in this species and that sex ratio distortions were found to occur even in wild populations of *P. lethostigma* (Honeycutt et al., 2019), the availability of a sex-determining gene could be instrumental for research on the impacts of global warming/climate change on natural populations of Japanese flounder. We designed this study with the ultimate goal of unravelling the genotypic sex determination mechanism in *P. olivaceus*. For this, information on sex-linked microsatellite markers and genome data were combined and used to identify a candidate sex-determining gene in *P. olivaceus*. The association between the candidate gene and phenotypic sex was investigated in farmed and wild individuals and its mRNA expression pattern during sex differentiation was analyzed.

## Materials and methods

### Source of fish used in this study

To identify sex-linked microsatellite markers, we generated a family (family-A) using a gynogenetic female (A-female: XX), produced by retention of the second polar body (meiotic gynogenesis), and a normal male (A-male: XY), derived by crosses between normal diploid fish at the Tottori Prefectural Fisheries Experimental Station (Tottori Prefecture, Japan). Larvae were treated with a low dose (0.3 μg/g of food weight) of estrogen (estradiol-17*β*; E_2_) from 41 to 70 dah (days after hatching) when they were an average total length of 20–50 mm. The purpose of the estrogen treatment was to inhibit spontaneous female-to-male sex reversal, as was described previously (Yamamoto, 1995; Yamamoto, 1999). The larvae were reared at a constant water temperature of 20°C. Nine months after hatching, the sex of the progeny was determined by visual inspection of the gonads. The proportion of phenotypic females and males was 46.2% (*n* = 36) and 53.8% (*n* = 42), respectively. A genetic map was constructed by genotyping the parents and their F1 offspring.

To fine map the sex-determining locus, we generated another family (family-B) by crossing a sex-reversed female (B-female: XY) and a sex-reversed male (B-male: XX), both of which were produced by hormone treatment and screened using the microsatellite markers *Poli30MHFS* and *Poli31MHFS*. The sex of progeny was determined by visual inspection of the gonads at 8 months of age. A total of 38 family-B individuals (25 males and 13 females) was used for the mapping. We used sex-reversed individuals because of the different rates of recombination between females and males, as described previously (Sakamoto et al., 2000; Castaño-Sánchez et al., 2010); females have higher rates of recombination near centromeres, whereas males have higher rates of recombination near telomeres. We expected that a linkage analysis using family-A and family-B families would allow a more accurate characterization of the sex-determining locus in *P. olivaceus*.

For the association analysis between the presence of the Y-specific *amh* gene (hereafter *amhy*) and phenotypic sex, we used wild-caught juveniles from the estuarine area of the Yurugawa River (Maizuru Bay, Japan). The juveniles were more than 50 mm (standard length) and supposedly had their phenotypic sex already determined in the wild. They were collected by seine net and reared for one more year at the National Research Institute of Aquaculture, Fisheries Research Agency (Oita, Japan), where the gonadal sex of all the individuals was determined by visual inspection under a stereomicroscope.

For the genetic analysis, genomic DNA was extracted from the caudal fin of each individual using either phenol/chloroform or a DNeasy Blood and Tissue kit (Qiagen, Hilden, Germany).

### Rearing conditions of experimental fish

Samples for the gene expression analysis were obtained from a single pair cross, which was selected due to the presence of a single-nucleotide polymorphism (SNP) in exon 2 of the candidate sex-determining gene (see description in the sequencing section). Egg incubation and larvae rearing were conducted in UV-treated sea water at 18°C for up to 100 dah under a constant light cycle (16-h light/8-h dark). Larvae/juveniles were measured and sampled every five or 10 days between 20 and 80 dah (Supplementary Table S1). The remaining fish were sampled and their phenotypic sex was determined by gonadal histology (100 dah). Caudal fins were fixed in ethanol for sex genotyping and trunks were fixed either in RNALater™ (Ambion Inc., Austin, United States) or 4% paraformaldehyde for expression analysis.

### Genotyping by microsatellite markers and linkage analysis of sex-determining locus

Microsatellite genotyping was performed in a 10-μl reaction volume containing 0.2 pmol/μl of unlabeled primer and 0.03 pmol/μl of end-labeled with [γ-^33^P]ATP using T4 polynucleotide kinase, plus 1× buffer, 0.2 mM dNTP, 1% bovine serum albumin, 0.02 U of *Taq* DNA polymerase, and 50 ng template DNA. A specific annealing temperature was used for each microsatellite marker. The PCRs were run on a GeneAmp^*®*^ PCR System 9700 (Applied Biosystems, Foster City, United States) under the following conditions: initial denaturation at 95°C for 2 min, followed by 35 cycles of 30 s at 95°C, 1 min at the annealing temperature, 1 min at 72°C, and final extension of 3 min at 72°C. The amplification products were mixed with 10 μl of loading buffer (95% formamide, 10 mM EDTA, 0.05% bromophenol blue, and xylene cyanol), denatured for 10 min at 95°C, and quickly cooled on ice. Then, 2 μl of each sample was loaded onto a 6% denaturing polyacrylamide gel (19:1 ratio acrylamide:bisacrylamide). After electrophoresis, the gels were dried on a standard gel drier for 30 min and exposed to imaging plates (FUJIFILM, Tokyo, Japan) overnight. The imaging plates were scanned with a Bio-image Analyzer, BAS1000 (FUJIFILM).

The linkage analysis to identify the sex-determining locus of Japanese flounder was carried out in three steps. In step 1, we analyzed the segregation of paternally inherited alleles of 63 microsatellite markers in 44 individuals (22 males and 22 females) of family-A to determine whether they were sex linked. These markers were selected to represent all linkage groups (LGs) of the genetic linkage map of Japanese flounder (Castaño-Sánchez et al., 2010). We analyzed the segregation of paternally inherited alleles of all the markers to determine whether they were sex linked. The linkages between the genotypes at each locus and phenotypic sex were tested using the Map Manager QT software program (Manly and Olson, 1999). Markers with LOD scores above 3.0 were considered potential sex-linked markers. In step 2, we used the potential sex-linked markers together with other markers that were mapped to the same LG to genotype all the individuals of family-A (42 males and 36 females). In step 3, for the fine mapping of the sex-determining locus, we used 59 markers on the same LG (LG 9) to genotype individuals of family-B (25 males and 13 females). Maps were drawn using MapChart v2.0 (Voorrips, 2002).

### Sequence analysis of scaffolds containing sex-linked microsatellite markers

We constructed the draft genome of *P. olivaceus* captured from the wild population of Wakasa Bay (Kyoto, Japan) by whole-genome shotgun assembly using Illumina short reads and PacBio long reads. The genome assembly has been deposited in the DDBJ database under accession numbers BRVK01000001–BRVK01002790.

The sex-linked microsatellite markers were searched against our draft scaffolds of the *P. olivaceus* genome using BLASTN (Altschul et al., 1990). Then, the sequences of the aligned scaffolds were analyzed using GENSCAN (Burge and Karlin, 1997) to predict the genes. The potential roles of the identified genes were inferred based on the UniProt database annotations (Bateman et al., 2021).

### Genome resequencing and screening for sex-linked SNPs

The genomes of 17 wild-caught *P. olivaceus* individuals were sequenced on an Illumina HiSeq X platform with 150-bp paired-end reads. The library construction and sequencing were conducted by BGI (Shenzhen, China). Low quality sequences were trimmed off using Trimmomatic v3.6 (Bolger et al., 2014) and mapped against the *amh*-containing scaffolds (786.920 bp) as reference using BWA-mem v0.7.12 (Li and Durbin, 2009) with default parameters. SNP detection was performed using Samtools mpileup (Garrison and Marth, 2012) with the following parameters: minimum coverage = 10, maximum coverage = 50. After removing mutations derived from low quality sequences using Vcffilter of Vcflib software, the high precision polymorphisms were filtered using VCFtools with parameters mac7, max0mac7, and max-missing-count 0 (Danecek et al., 2011).

To detect SNPs in the *amh* gene, wild-caught *P. olivaceus* (20 females and 8 males) were analyzed by direct sequencing. PCR fragments were amplified using specific primers and sequenced on an ABI PRISM 3100 capillary sequencer (Applied Biosystems, Foster City, United States) using the BigDye Terminator method. Sequences were analyzed using GENETYX v12.0 (GENETYX, Tokyo, Japan).

### Comparative sequence analysis of amh in XX and XY *P. olivaceus*

To amplify the *amh* gene sequences, including exons, introns, and the 5′- and 3′-flanking regions, DNA was extracted from the caudal fin of XX and XY fish using a Qiagen DNA Extraction Kit (Qiagen, Hilden, Germany). PCRs were performed in a total volume of 10 μl with Ex Taq polymerase (Takara Bio, Shiga, Japan) and the primers described in Supplementary Table S2. Amplification was performed in a Thermocycler T professional BASIC 96 Gradient (Biometra GmbH, Goettingen, Germany). Fragments were excised from the gel, purified, and cloned into a pGEM-T easy vector (Promega, Corporation, Madison, WI). Sequencing was performed as described in the previous section.

### Amplification of 5′ and 3′ untranslated regions by RACE-PCR

To isolate the 5′ and 3′ untranslated regions of the Y-specific *amhy* gene, gonads of a genetic male larva (55 dah) expressing *amh* (previously assessed) were used. To isolate the 5′ and 3′ untranslated regions of the X-specific *amh* gene (*amhx*), ovary tissues from an adult XX female were used. The extracted RNA was reverse transcribed using a Clontech SMART rapid amplification of cDNA ends (RACE) Amplification Kit (BD Biosciences, Franklin Lakes, United States) and used for PCRs with gene-specific primers (Supplementary Table S3) following the manufacturer’s instructions. The PCR-amplified fragments were cloned and sequenced, as described in the previous section.

### Sex genotyping by 5′ upstream region amplification of the *amh* gene

Caudal fin samples were treated with 50 mM NaOH for 5 min at 95°C, equilibrated with Tris-HCl (pH 8.0), and briefly centrifuged to isolate the DNA template. The aqueous phase containing DNA was used for genotyping using KOD Fx Neo (TOYOBO, Osaka, Japan) and primers amhFw1 5′-AAG​TTC​AGT​TCA​GTT​GCA​CAG​C-3′ and amhRv1 5′-CTT​GAC​AAA​CAG​GGC​ATG​AAT​A-3′ under the following conditions: initial denaturation at 94°C for 2 min, followed by 35 cycles of 30 s at 95°C, 30 s at 64°C, and 1 min at 68°C, and final elongation of 3 min at 68°C. The amplified products were electrophoresed in 1% agarose gel and visualized by ethidium bromide staining. Samples with two bands (931 bp and 687 bp) were scored as genotypic males (XY) and those with a single band (931 bp) were scored as genotypic females (XX).

### Expression analysis by qRT-PCR during sex differentiation

Total RNA was extracted from larval trunks using an RNeasy mini kit (QIAGEN K.K., Tokyo, Japan) following the manufacturer’s instructions. RNA samples (1 μg) were treated with deoxyribonuclease I amplification grade (Invitrogen) and reverse transcribed using SuperScript III RNase H-Reverse Transcriptase (Invitrogen) with oligo(dT)12-18 following the manufacturer’s instructions. The quantitative real-time PCRs (qRT-PCRs) were performed in 20-µl reaction volumes using either Premix Ex Taq™ and specific TaqMan minor-groove binder (MGB) probes for both *amhy* and *amhx* or TB Green^®^ Premix Ex Taq™ for *cyp19a1a* and *amhrII* (both from Takara Bio Inch, Shiga, Japan). Amplification was done using 1 µl of first strand cDNA (approximately 25 ng) and 5 pmol of each primer (Supplementary Table S3) in a StepOne Plus Real-Time PCR system (Applied Biosystems, Foster City, United States). Transcript abundance was quantified using the standard curve method with four dilution points and normalized against *elf1a* values.

### Histological determination of sex ratios and localization of *amh* transcripts by *in situ* hybridization

Larval trunks were collected at the end of the experimental period and fixed in 4% paraformaldehyde solution overnight, dehydrated in an ascending ethanol series, and stored in absolute ethanol. Samples were embedded in Paraplast Plus (McCormick, St. Louis, United States), sectioned transversally at a thickness of 6 μm, and mounted on glass slides. Sections were stained with hematoxylin-eosin and observed under a microscope for determination of gonadal sex.

*In situ* hybridization was performed in larvae collected before (35 dah) and after (100 dah) the onset of histological differentiation of the gonads using the procedures described previously (Sarida et al., 2019). The probe was synthesized using the same primers as those used in a previous study (Yoshinaga et al., 2004). Hybridization was conducted in the transverse sections overnight at 63°C. NBT/BCIP was used for signal detection in accordance with the manufacturer’s recommendations (Roche Diagnostics, Basel, Schweiz).

### CRISPR-Cas9-mediated loss-of-function of the *amh* gene

Synthetic CRISPR RNAs (crRNAs) and trans-activating crRNA (tracrRNA) were obtained from Fasmac Co., Ltd. (Kanagawa, Japan). The sequences of the two crRNAs for *amh* were Target 1 5′-CUA​UCU​GCA​GCU​CGU​AGG​UAg​uuu​uag​agc​uau​gcu​guu​uug-3′ and Target 2 5′-AAU​UAA​AAA​GCA​CAU​UUU​Gag​uuu​uag​agc​uau​gcu​guu​uug-3′. These sequences were designed on the basis of Sawamura et al. (Sawamura et al., 2017). Two crRNAs (250 ng/μl), tracrRNA (500 ng/μL), and Cas9 protein (750 ng/μl; Toyama, Nippongene) were mixed and immediately injected into fertilized eggs using a microinjector (IM-9B, Narishige, Tokyo, Japan). Two crRNAs and Cas9 protein without tracrRNA were injected as experimental controls.

The artificial fertilization, microinjection, and rearing of the experimental fishes were performed at the Nansei Field Station, Japan Fisheries Research and Education Agency. Eggs and sperm from three-year-old females and males were used. They were mixed in a plastic beaker sampler and activated with sterilized seawater (18°C) for artificial fertilization. At 200 dah, the gonads were isolated from each fish; one lobule was processed for histological analysis and the other was used for sex genotyping. All the fish were sampled after ensuring that they had been completely euthanized by an overdose of 2-phenoxyethanol (Wako Chemicals).

### Sequence analysis of mutations

Genomic DNA was extracted from the isolated gonads using ISOGEN^®^ (Nippongene) according to the manufacturer’s instructions. Genomic PCRs were carried out using AmpliTaq Gold^®^ (Applied Biosystems) with the following *amh*-specific primers: Forward 5′-TTT​CTT​CTC​CTG​AAG​GCC​C-3′ and Reverse 5′-ATT​AGC​TGT​CAC​AGC​AGC​AG-3′. The PCR conditions were as follows: pre-heating at 95°C for 2 min, followed by 35 cycles at 95°C for 15 s, 59°C for 30 s, 72°C for 2 min, and final extension at 72°C for 5 min. The amplified PCR fragments were subcloned using a TA PCR Cloning Kit (BioDynamics Laboratory Inc., Tokyo, Japan), then sequenced as described previously.

### Statistical analysis

The significance of differences between groups was determined by one-way ANOVA followed by the Tukey test for gene expression using GraphPad Prism v6.0 (GraphPad Software, San Diego, United States). Differences were considered as statistically significant at *p* < 0.05.

## Results

### Linkage analysis and fine mapping of sex-determining locus

**FIGURE 1:**
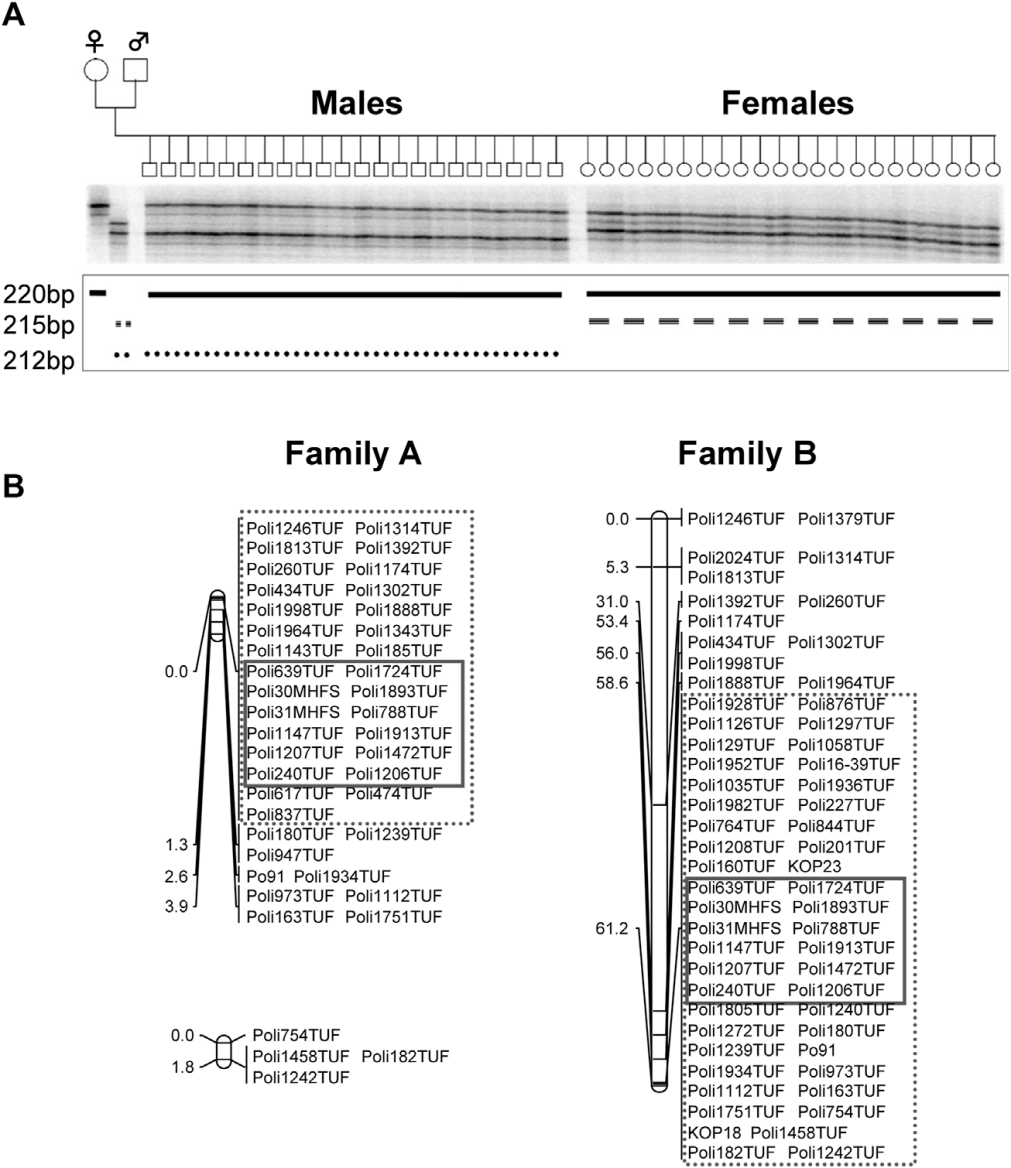
Autoradiograph of Poli185TUF in JF9 in the A-family at step 1 **(A)**. The lower 212 bp allele from the male parent was the unique allele inherited by the males of this family. JF9-male linkage map in Family A and JF9 linkage map in Family B **(B)** Map distances between markers are shown in centimorgans (cM). 35 markers in the enclosure with a dotted line were mapped close to sex-determining locus in A-family, while 46 markers in the enclosure with a dotted line were mapped in family **(B)**. Common 12 markers in the enclosure with a straight line were detected as closed markers to the sex-determining locus.

The sex-determining locus was identified in LG 9 (Figure 1A and Table 1) in family-A. We used the sex-linked markers identified in step 1 of the linkage analysis and additional microsatellite markers in LG 9 in all individuals of family-A and found 29 markers that were perfectly linked with the sex-determining locus with no recombination in family-A. In the fine mapping using family-B, we identified 46 markers that were tightly linked with the sex-determining locus with no recombination. Among them, we detected 12 markers that were perfectly linked with the sex-determining locus and were commonly shared by both families (Figure 1B and Table 2).

**TABLE 1:** Segregation of paternally inherited alleles of 63 microsatellite markers in 44 individuals (22 males and 22 females) of family A. The marker in bold represent the sex-determining locus.

Linkage group	Locus	*P*	LOD score	Linkage group	Locus	*P*	LOD score
1	Poli110TUF	0.32	0.22	11	Poli174TUF	0.01	1.41
	Poli41MHFS	0.32	0.22		Poli176TUF	0.12	0.52
	Poli9-67TUF	0.13	0.50		Poli132TUF	0.06	0.74
2	Poli23TUF	0.54	0.09	12	Poli38MHFS	0.76	0.02
3	Poli13TUF	0.07	0.74		Poli179TUF	0.35	0.20
	Poli188TUF	0.76	0.02		Poli149TUF	0.36	0.17
	Poli24MHFS	0.19	0.37	13	Poli145TUF	0.22	0.33
	Poli13MHFS	0.54	0.09		Poli17MHFS	0.22	0.33
	Poli18-51TUF	0.07	0.74		Poli204TUF	0.54	0.09
4	Poli148TUF	0.22	0.33	14	Poli-RC47-TUF	0.53	0.09
	Poli128TUF	0.54	0.09		Poli120TUF	0.22	0.33
	Poli19TUF	0.75	0.02	15	Poli9-8TUF	1.00	0.00
	Poli14MHFS	0.76	0.02		Poli121TUF	0.76	0.02
	Poli142TUF	0.75	0.02		Poli-RC15-35-TUF	0.22	0.33
5	Poli43TUF	0.76	0.02	16	Poli114TUF	0.22	0.33
	Poli169TUF	0.75	0.02		Poli199TUF	0.76	0.02
	Poli151TUF	0.55	0.09	17	Poli127TUF	0.75	0.02
6	Poli190TUF	0.21	0.35		Poli11TUF	0.76	0.02
	Poli143TUF	0.36	0.17	18	Poli147TUF	0.36	0.17
	Poli107TUF	0.22	0.33		Poli16-79TUF	1.00	0.00
7	Poli18-55TUF	0.36	0.17	19	Poli108TUF	1.00	0.00
	Poli113MHFS	1.00	0.00		Poli186TUF	0.22	0.33
	Poli50TUF	1.00	0.00	20	Poli123TUF	0.76	0.02
8	Poli202TUF	0.15	0.43		Poli9-58TUF	0.00	0.00
	Poli126TUF	0.36	0.17	21	Poli7 MHFS	0.76	0.02
9	Poli182TUF	0.54	0.09		Poli109 MHFS	0.22	0.33
	**Poli185TUF**	**0.00**	**13.25**	22	Poli2TUF	0.06	0.78
	Poli180TUF	0.00	11.17	23	Poli150TUF	0.55	0.09
10	Poli144TUF	0.55	0.09		Poli18-42TUF	0.36	0.17
	Poli13-2TUF	0.55	0.09		Poli56TUF	0.09	0.61
	Poli37MHFS	0.76	0.02		Poli-RC27-TUF	0.02	1.15
				24	Poli183TUF	0.36	0.17

**TABLE 2:** Detailed list of scaffolds blasted against 12 sex-linked microsatellite markers.

Marker name	Scaffold #	Identity	Start	End	E-value	Accession number
Poli1206TUF	318	97.235	193,315	193,099	5.7E-99	BRVK01000318
Poli240TUF	717	97.561	80,385	80,917	0	BRVK01000717
Poli1472TUF	801	93.269	105,926	106,237	3.7E-122	BRVK01000801
Poli1207TUF	126	94.86	50,689	50,902	7.24E-88	BRVK01000126
Poli1913TUF	346	100	672,910	672,650	5.3E-135	BRVK01000346
Poli1147TUF	362	98.319	113,310	113,547	3.8E-116	BRVK01000362
Poli788TUF	410	97.674	297,718	297,289	0	BRVK01000410
Poli31MHFS	752	98.374	237,106	237,228	1.59E-54	BRVK01000752
Poli1893TUF	274	98.106	76,773	76,514	3.3E-127	BRVK01000274
Poli30MHFS	no hits					-
Poli1724TUF	301	92.808	82,166	81,890	4.7E-111	BRVK01000301
Poli639TUF	970	94.407	174,735	174,147	0	BRVK01000970

### Sequence analysis of scaffolds containing sex-linked microsatellite markers

The *P. olivaceus* genome assembly yielded 2,790 scaffolds longer than 1,000 bp. The longest scaffold was 1.81 Mb with N50 of 357.0 kb, the mean scaffold length was 217.0 kb, and the total size of the assembly was 605.6 Mb. The 12 sex-linked microsatellite markers were distributed in 11 of the scaffolds (Table 2). A total of 181 genes were predicted in these scaffolds based on the UniProt database annotations (Supplementary Table S4). Among them, we selected the *amh* gene because of its known involvement with male sex determination in some teleosts.

### Linkage analysis between SNPs and the sex phenotype

We detected 69 SNPs in the *amh* gene and 15 of them were highly associated with maleness (Table 3). The in-depth analysis of coding and non-coding regions of the *amh* gene in XX and XY individuals detected nine SNP markers in exons 2, 3, 4, 6, and 7, but none of them were synonymous substitutions (Table 4).

**TABLE 3:** Localization of 15 SNPs that showed association with sex in relation with the predicted genes from the 786.920 bp scaffold. Gene #101 corresponds to the *amh* gene.

SNP type		Gene #	Region	Type of substitution	Position in the scaffold
XX	XY				
T/T	T/A	83	Intron	-	47,837
C/C	C/G	96–97	Intron	-	228,359
T/T	T/A		Intron	-	229.620
T/T	T/A	98	Intron	-	234,671
C/C	C/T	100	Intron	-	242,211
C/C	C/T		Intron	-	242,984
A/A	A/T	101	Intron	-	243,573
G/G	G/A		Exon	Synonymous	244,664
C/C	C/T		Exon	Synonymous	245,185
T/T	T/A		Intron	-	245,289
A/A	A/T		Intron	-	247,694
T/T	T/C	102	Intron	-	255,206
G/G	G/A		Exon	Synonymous	257,160
T/T	T/A		Intron	-	258,300
G/G	G/A		Intron	-	260,102

**TABLE 4:** Detailed characterization of SNPs detected in the coding regions of *amh* gene in XX and XY genotypes.

Region		Exon 2		Exon 3	Exon 4	Exon 6	Exon 7					
							CDS		3̓ UTR			
SNP ID		#01	#02	#03	#04	#05	#06	#07	#08		#09	
Position from start codon (bp)		289	493	631	924	1445	2014	2034	2544		2546	
Females (XX)	F01	C/C	T/T	C/T	C/C	G/G	C/C	C/C	G/G		A/A	
	F02	C/C	T/T	C/T	C/T	G/G	C/C	C/C	G/G		A/A	
	F03	C/C	T/T	C/T	DEL	G/A	C/C	C/C	G/G		A/A	
	F04	C/C	T/T	C/C	C/C	G/G	C/C	C/C	G/G		A/A	
	F05	C/C	T/T	C/C	C/C	G/G	C/C	C/C	G/G		A/A	
	F06	C/C	T/T	C/C	C/C	G/G	C/C	C/C	G/G		A/A	
	F07	C/C	T/T	C/C	C/C	G/G	C/C	C/C	G/G		A/A	
	F08	C/C	T/T	C/C	C/C	G/G	C/C	C/C	G/G		A/A	
	F09	C/C	T/T	C/C	C/C	G/G	C/C	C/C	G/G		A/A	
Males (XY)	M01	C/T	T/G	C/T	C/T	G/A	C/T	C/T	G/T		A/T	
	M02	C/T	T/G	C/T	C/T	G/A	C/T	C/T	G/T		A/T	
	M03	C/T	T/G	C/T	C/T	G/A	C/T	C/T	G/T		A/T	
	M04	C/T	T/G	C/T	C/T	G/A	C/T	C/T	G/T		A/T	
	M05	C/T	T/G	C/T	C/T	G/A	C/T	C/T	G/T		A/T	
	M06	C/T	T/G	C/T	C/T	G/A	C/T	C/T	G/T		A/T	
	M07	C/T	T/G	C/T	C/T	G/A	C/T	C/T	G/T		A/T	
	M08	C/T	T/G	C/T	C/T	G/A	C/T	C/T	G/T		A/T	

### Size polymorphism in the promoter regions of the *amh* gene

**FIGURE 2:**
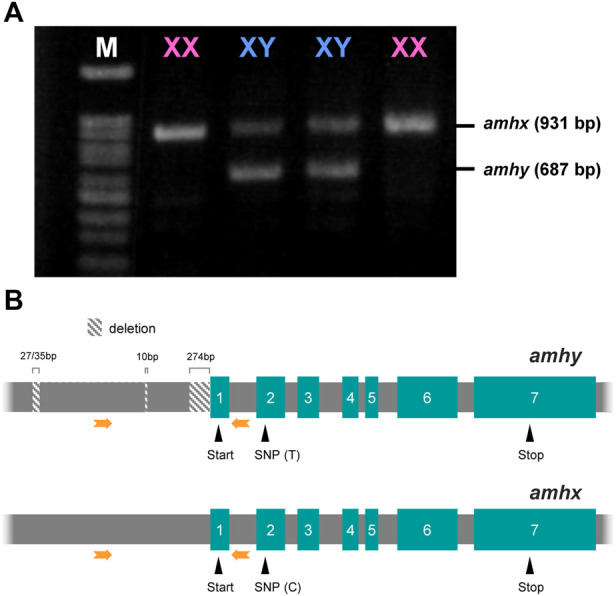
Amplification pattern of 5′ upstream region and the structure of *amhy* and *amhx* genes in *P. olivaceus*. **(A)** Agarose gel electrophoresis of *amhx* and *amhy* 5′ upstream region amplified by PCR in XX and XY individuals, phenotypic females and males, respectively; *amhy* band is shorter than *amhx* due to deletions in the presumptive promoter. **(B)** Structures of *amhy* and *amhx* genes showing the position of three *amhy*-specific deletions in the 5′ upstream region, both start and stop codons, and a SNP in the second exon; thymine (T) and cytosine **(C)** in exon 2 were highly linked to *amhy* and *amhx*, respectively. Green boxes represent the exons with the respective number and grey boxes in between represent the introns. Orange arrows indicate the position of primers used in **(A)**

Amplification of the region 5′ upstream of the start codon and gel electrophoresis of the products showed that there was a sex-specific polymorphism in the banding pattern; genetic females had one band and genetic males had two bands. One of the two bands in the males was the same size as the band in the females; the other band was slightly shorter (Figure 2A). The sequence analysis showed that the male-specific band had deletions in three segments, one of 274 bp, one of 10 bp, and one of 27 bp or 32 bp at positions −14 bp, −449 bp, and −972 bp, respectively (the positions correspond to the *amhx* gene sequence, which was used as the reference; Figure 2B). The sequences of the coding regions of the *amhy* and *amhx* genes were almost identical, indicating the presence of two alleles for *amh* that differ in the presumptive promoter and that theoretically encode identical proteins because the SNPs detected in some of the exons were synonymous substitutions. No other *amh* homologs were identified in other chromosomes based on searches against the *P. olivaceus* genome database provided in this study.

### Association analysis between *amhy* and the male phenotype

The association analysis using females and males for which the genotypic sex was inferred by the presence/absence of *amhy* showed complete association with phenotypic sex and genotype in captive-reared individuals used to identify the microsatellite markers. Complete association was also found in a wild-caught population from Maizuru Bay, the Sea of Japan; 20 females where negative for *amhy* and the eight males were *amhy*-positive. Considering that *P. olivaceus* has an XX-XY sex determining system, it can be surmised that the first allele is on the Y chromosome and its counterpart is on the X chromosome. For this reason, the lighter band was named *amhy* (Y chromosome-linked *amh*), whereas the first, upper band was named *amhx* (X chromosome-linked *amh*)*.*

The synonymous SNP that was found in the 39^th^ bp of exon 2, showed a high but not full association with two of the *amhy*-specific deletions; 94.74% of fish had the nucleotide T in *amhy* and C in *amhx*.

### Expression analysis of *amhy* during sex differentiation

**FIGURE 3:**
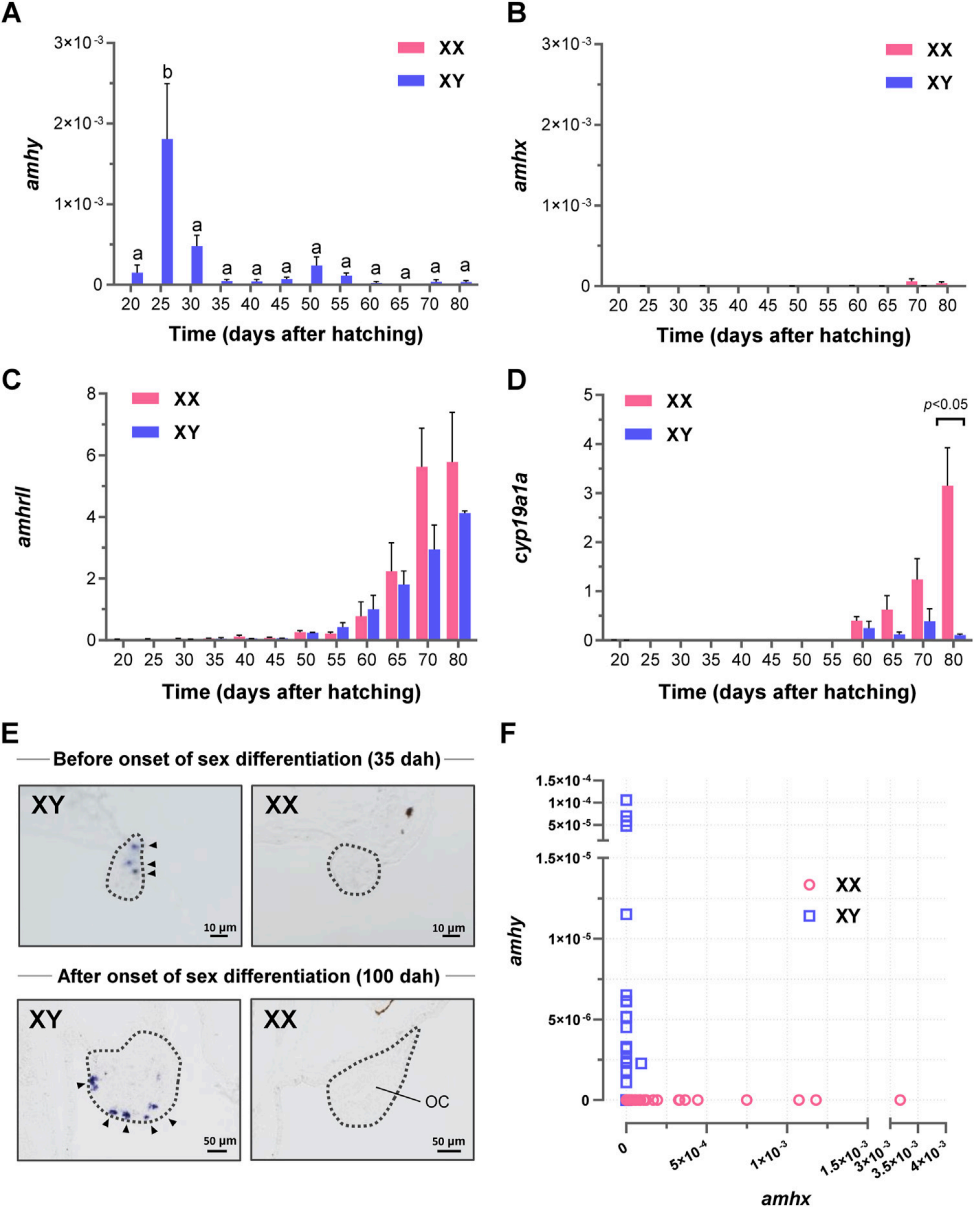
Expression analysis of *amhy, amhx, amhrII*, and *cyp19a1a* mRNA during sex differentiation in *P. olivaceus*. Expression profiling by qRT-PCR in larvae trunks showed **(A)** *amhy* expression only in XY genotypes with a peak at 25 dah. **(B)** *amhx* was almost undetectable during the same period; weak expression was detected at 70 and 80 dah. **(C,D)** Quantitative analysis of *amhrII* and *cyp19a1a* mRNAs during the same period; sexually dimorphic expression was detected only for *cyp19a1a*, at 80 dah XX samples. **(E)** Spatial localization of *amh* riboprobe restricted to XY gonads before and after the onset of sex differentiation; at 100 dah, signals were recognized at the ventral edge of the XY gonad, where germ cells are distributed to form cysts. In XX gonads, which had a clear ovarian cavity (OC), no detectable signals of *amh* were found. **(F)** the relationship between *amhy* and *amhx* expression in XY in relation to *amhx* in XX; although absolute expression values were generally lower than *amhy*, for some reason more XX individuals were expressing *amhx* than XY. Differences were considered as significant for *p* < 0.05 by One-Way ANOVA with Turkey post-test.

Expression analysis by qRT-PCR detected *amhy* transcripts from 20 to 80 dah in the XY samples with a peak at 25 dah (Figure 3A), whereas *amhx* mRNA expression was very low or undetectable during the same period in both genotypes (Figure 3B). The expression of *amhrII* was detected from 25 dah and increased gradually from 55 dah onwards (Figure 3C), and that of *cyp19a1a* was upregulated from 60 dah and showed increasing expression from 65 dah in XX samples (Figure 3D). Localization *amh* mRNAs by *in situ* hybridization showed positive signals in gonads before and during gonadal sex differentiation only in the XY genotype (Figure 3E). At 35 dah, the signals were detected in presumptive Sertoli cells, whereas at 100 dah, when clear characteristics of testis differentiation can be detected, strong signals were observed in presumptive Sertoli cells surrounding cysts of germ cells at the ventral side of the gonad. Although, the expression of *amhx* was detected by qRT-PCR in some samples, the levels were extremely low compared with the expression levels of *amhy* (Figure 3F), which suggests that the *in situ* hybridization signals detected in XY gonads most probably represent the expression of *amhy*.

Analysis of the phenotypic sex at the end of the rearing experiment (100 dah) by gonadal histology in 18 larvae showed that individuals with differentiating ovaries were all *amhy*-negative XX (*n* = 8) and those with a differentiating testis were all *amhy*-positive XY (*n* = 10).

### Generation of *amh*-mutant Japanese flounder using the CRISPR-Cas9 system

**FIGURE 4:**
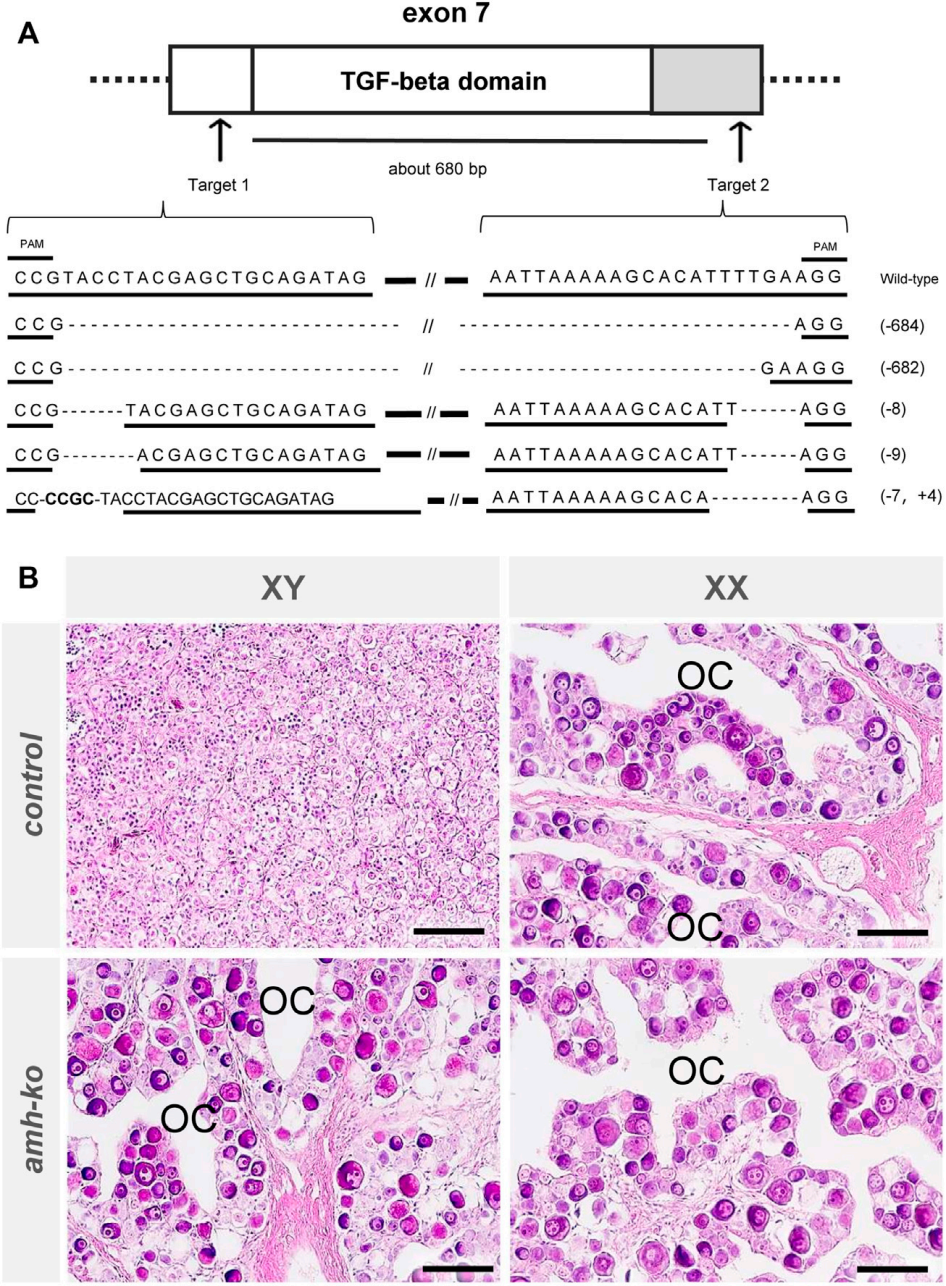
Production of *amh* mutant Japanese flounder using CRISPR/Cas9 system. **(A)** Schematic positions of crRNAs targeted on *amh* locus, showing exons (white boxes), untranslated regions (gray boxes), introns (horizontal lines) and target sequences of the crRNAs with CRISPR/Cas9 system (arrows). The numbers between target sequences indicate approximate deletion size by CRISPR/Cas9 system. Sequences of wild-type *amh* and typical mutation of *amh* mutant with the rate in two target regions. The underlined sequences indicate the two crRNA target sequences. The horizontal lines and dashed line indicate wild-type sequences and deletions, respectively. Slashes indicate omission of long sequences. The number in parentheses indicates the size of deletion (-) or insertion (+). **(B)** Histological images of gonads in wild-type and *amh* mutant Japanese flounder at 200 days after hatching (dah). In controls, genetic male gonad showed typical testis containing many spermatogonia, and genetic female gonad showed typical ovary harboring many oocytes and an ovarian cavity (OC), which exhibits morphological characteristics of the ovary. In *amh* mutants, the genetic male and female gonads showed typically normal ovaries containing many oocytes and an ovarian cavity. Scale bars, 50 µm.

To elucidate the roles of a*mh* in Japanese flounder, we produced *amh*-mutant flounder using the CRISPR-Cas9 system. We selected two target sequences to obtain wide-range deletion mutations that deleted the functional regions of *amh* (Sawamura et al., 2017). The crRNA target sequences were designed in exon 7 of the *amh* gene, which encodes a conserved TGF-beta domain at its 3′ end. We expected that by deleting the approximately 680-bp region the encoded Amh protein would lack function (Figure 4A). The two crRNAs were co-injected into fertilized eggs, and hatchlings were reared until 200 dah to investigate the mutation efficiency. The amplicon DNA fragments of the *amh*-mutants were examined by sequence analysis, which showed 682 bp or 684 bp deletions between the two target sequences and some indel mutations in both target sequences (Figure 4A). The wide-range deletion efficiency in genetic male fish was 81.3%, 75.0%, and 75%, and the efficiency in genetic female fish was 75.0% and 71.4% (Table 5).

**TABLE 5:** Genotypic and phenotypic sexes in juveniles of *P. olivaceus* and the types and frequencies of *amh* mutations in each individual.

Genotype	Genotypic sex	Phenotypic sex (gonad)	Number of clones sequenced	Types of mutation			Rate of long deletion (%)
				Long deletion	Shorts indels	No mutation	
Wild-type	XY	♂ (Testis)	4	0	0	4	0
	XX	♀ (Ovary)	4	0	0	4	0
*amh* mutant	XY	♀ (Ovary)	16	13	3	0	81.3
	XY	♀ (Ovary)	16	12	4	0	75.0
	XY	♀ (Ovary)	16	12	4	0	75.0
	XX	♀ (Ovary)	8	6	2	0	75.0
	XX	♀ (Ovary)	7	5	2	0	71.4

### Loss of Amh function causes male-to-female sex reversal in *P. olivaceus*

To investigate the phenotypes of the *amh*-mutants in *P. olivaceus*, we performed histological analysis of the gonads of wild-type (control) and *amh*-mutant Japanese flounder at 200 dah. Histological observations of the gonads showed that in the controls, all the genetic male gonads were typical testes containing many spermatogonia (Figure 4B), and all the genetic female gonads were typical ovaries harboring many oocytes and an ovarian cavity. In the *amh*-mutants, all the genetic male and female gonads were typical normal ovaries containing many oocytes and an ovarian cavity. These results indicate that the loss of Amh function caused male-to-female sex reversal in *P. olivaceus*.

## Discussion

The number of genome sequencing projects in teleost fish has increased in recent years, and reports of genetic switches of sex determination have increased for many other fish species (Hattori et al., 2020). In this study, we combined sex-linked microsatellite marker information and genome database analysis, and selected genes related to gonadal differentiation from a list of 181 predicted genes. The detailed comparative sequence analysis of the predicted genes identified a male-specific *amh* gene (named as *amhy*) in the sex-linked locus. Although the coding region of *amhy* was identical to that of *amhx*, except for one synonymous SNP, the presumptive *amhy* promoter differed from that of the female-specific *amhx* due to three deletions within a 1-kb fragment. These deletions are seemingly associated with the early expression of *amhy* in XY, which occurs before the onset of histological sex differentiation. These characteristics place *amhy* as the master sex-determining gene of the Japanese flounder *Paralichthys olivaceus*.

The longest deletion in the presumptive *amhy* promoter corresponds to a region close to the start codon (at position −14 bp) and the other two deletions are located in a further upstream region. Which of these deletions are responsible for the early expression of *amhy* needs further examination by promoter and/or functional analysis. Nevertheless, because the coding regions of *amhy* and *amhx* are almost identical, it seems reasonable to assume that modifications in their regulatory regions are responsible for the different expression profile of these two genes. Some sex-determining genes reported previously have differences in the coding nucleotide and amino acid sequences, and these differences are associated with genotypic sex determination in *Takifugu rubripes amhrII* (Kamiya et al., 2012) and *Seriola quinqueradiata hsd17b1* (Koyama et al., 2019). The pattern of *amhy* expression in *P. olivaceus* is similar to that of sex-determining genes in the medaka *Oryzias luzonensis sox3* ^*Y*^ (Takehana et al., 2014) that differ by only a 9-bp deletion in the promoter region.

Although many sex-determining genes have been described in teleost fish (Hattori et al., 2020), there seems to be a high likelihood that *amh* will take over the position of sex-determining gene (Bej et al., 2017; Hattori et al., 2019; Pan et al., 2021; Song et al., 2021), and this has been corroborated by this study. Besides the sex linkage, another characteristic shared by *amhy* genes of other species is their high expression in pre-Sertoli cells before the onset of histological sex differentiation, which is seemingly associated with changes in promoter regions. In *P. olivaceus*, we detected the expression of *amhy* from 20 dah with a peak at 25 dah. Although we did not assess the expression before 20 dah, 25 dah can be considered as relatively early (Kitano et al., 1999; Kitano et al., 2000) and much earlier than the upregulation of *cyp19a1a* in XX, which started to increase at 65 dah. The expression of *amhrII*, the receptor that is supposed to bind to *amh*, was also detected at 20 dah (although at low levels) and increased gradually thereafter. Interestingly, the expression of *amhx* was higher in XX than it was in XY genotypes. Although this may be because XX individuals have two copies of *amhx* and XY individuals only one, this expression pattern deserves further investigation considering that *amhx* was upregulated in XX fish during female-to-male sex reversal as was reported previously (Yamaguchi et al., 2010).

The development of ovaries in XY genotypes (i.e., male-to-female sex reversal) by disrupting the region of the *amh* (presumably *amhy*) gene that encodes the TGF-beta domain using CRISPR-Cas9 technique demonstrated that this gene is necessary for testicular formation in *P. olivaceus* genotypic males and indicated that its downstream function may be mediated by the TGF-beta domain. How widespread the *amhy* of *P. olivaceus* is among other closely-related groups needs further investigation, but the *amhy* gene of *P. olivaceus* shares high similarity with the *amhy* genes of other species that have been described so far, which may indicate recent evolution of *amhy* genes. Considering the sex-determining candidates *dmrt1* in tongue sole (Chen et al., 2014) and *sox2* in turbot (Martínez et al., 2021), the discovery of genes other than *amhy* seems to illustrate that genotypic sex determination in flatfishes in order Pleuronectiformes may involve a variety of sex-determining genes.

In conclusion, the present results support the view that *amhy* is the master sex-determining gene of Japanese flounder *Paralichthys olivaceus*. Further promoter analysis studies may help us to understand the importance of this gene and the transcription regulation behind its expression profile. The availability of a reliable marker of genotypic sex will be instrumental for understanding the interactions between genotypic and environmental sex determination from aquaculture and ecological perspectives.

## Data Availability

The datasets presented in this study can be found in online repositories. The names of the repository/repositories and accession number(s) can be found below: https://www.ddbj.nig.ac.jp/, BRVK01000001–BRVK01002790.

## References

[B1] Altschul S. F. Gish W. Miller W. Myers E. W. Lipman D. J. (1990). Basic local alignment search tool. J. Mol. Biol. 215, 403–410. 10.1016/S0022-2836(05)80360-2 2231712

[B2] Baroiller J. F. D’Cotta H. (2016). The reversible sex of gonochoristic fish: Insights and consequences. Sex. Dev. 10, 242–266. 10.1159/000452362 27907925

[B3] Bateman A. Martin M. J. Orchard S. Magrane M. Agivetova R. Ahmad S. (2021). UniProt: The universal protein knowledgebase in 2021. Nucleic Acids Res. 49, D480–D489. 10.1093/nar/gkaa1100 33237286 PMC7778908

[B4] Bej D. K. Miyoshi K. Hattori R. S. Strüssmann C. A. Yamamoto Y. (2017). A duplicated, truncated amh gene is involved in male sex determination in an old world silverside. G3(Bethesda) 7, 2489–2495. 10.1534/g3.117.042697 28611256 PMC5555456

[B5] Bolger A. M. Lohse M. Usadel B. (2014). Trimmomatic: A flexible trimmer for Illumina sequence data. Bioinformatics 30, 2114–2120. 10.1093/bioinformatics/btu170 24695404 PMC4103590

[B6] Burge C. Karlin S. (1997). Prediction of complete gene structures in human genomic DNA. J. Mol. Biol. 268, 78–94. 10.1006/jmbi.1997.0951 9149143

[B7] Castañeda Cortés D. C. Arias Padilla L. F. Langlois V. S. Somoza G. M. Fernandino J. I. (2019). The central nervous system acts as a transducer of stress-induced masculinization through corticotropin-releasing hormone B. Development 146, dev172866–10. 10.1242/dev.172866 30936180

[B8] Castaño-Sánchez C. Fuji K. Ozaki A. Hasegawa O. Sakamoto T. Morishima K. (2010). A second generation genetic linkage map of Japanese flounder (*Paralichthys olivaceus*). BMC genomics 11, 554. 10.1186/1471-2164-11-554 20937088 PMC3091703

[B9] Chen S. Zhang G. Shao C. Huang Q. Liu G. Zhang P. (2014). Whole-genome sequence of a flatfish provides insights into ZW sex chromosome evolution and adaptation to a benthic lifestyle. Nat. Genet. 46, 253–260. 10.1038/ng.2890 24487278

[B10] Danecek P. Auton A. Abecasis G. Albers C. A. Banks E. DePristo M. A. (2011). The variant call format and VCFtools. Bioinformatics 27, 2156–2158. 10.1093/bioinformatics/btr330 21653522 PMC3137218

[B11] Fernandino J. I. Hattori R. S. Moreno Acosta O. D. Strüssmann C. A. Somoza G. M. (2013). Environmental stress-induced testis differentiation: Androgen as a by-product of cortisol inactivation. Gen. Comp. Endocrinol. 192, 36–44. 10.1016/j.ygcen.2013.05.024 23770022

[B12] Garrison E. Marth G. (2012). Haplotype-based variant detection from short-read sequencing. arXiv.

[B13] Hattori R. S. Castañeda-Cortés D. C. Arias Padilla L. F. Strobl-Mazzulla P. H. Fernandino J. I. (2020). Activation of stress response axis as a key process in environment-induced sex plasticity in fish. Cell. Mol. Life Sci. 77, 4223–4236. 10.1007/s00018-020-03532-9 32367192 PMC11104976

[B14] Hattori R. S. Fernandino J. I. Kishil A. Kimura H. Kinno T. Oura M. (2009). Cortisol-induced masculinization: Does thermal stress affect gonadal fate in pejerrey, a teleost fish with temperature-dependent sex determination? PLoS One 4, e6548. 10.1371/journal.pone.0006548 19662094 PMC2717333

[B15] Hattori R. S. Murai Y. Oura M. Masuda S. Majhi S. K. Sakamoto T. (2012). A Y-linked anti-Mullerian hormone duplication takes over a critical role in sex determination. Proc. Natl. Acad. Sci. U. S. A. 109, 2955–2959. 10.1073/pnas.1018392109 22323585 PMC3286941

[B16] Hattori R. S. Somoza G. M. Fernandino J. I. Colautti D. C. Miyoshi K. Gong Z. (2019). The duplicated Y-specific amhy gene is conserved and linked to maleness in silversides of the genus Odontesthes. Genes 10, 679. 10.3390/genes10090679 PMC6770987 31491991

[B17] Hayashi Y. Kobira H. Yamaguchi T. Shiraishi E. Yazawa T. Hirai T. (2010). High temperature causes masculinization of genetically female medaka by elevation of cortisol. Mol. Reprod. Dev. 77, 679–686. 10.1002/mrd.21203 20653000

[B18] Honeycutt J. L. Deck C. A. Miller S. C. Severance M. E. Atkins E. B. Luckenbach J. A. (2019). Warmer waters masculinize wild populations of a fish with temperature-dependent sex determination. Sci. Rep. 9, 6527–6613. 10.1038/s41598-019-42944-x 31024053 PMC6483984

[B19] Ieda R. Hosoya S. Tajima S. Atsumi K. Kamiya T. Nozawa A. (2018). Identification of the sex-determining locus in grass puffer (*Takifugu niphobles*) provides evidence for sex-chromosome turnover in a subset of *Takifugu* species. PLoS One 13, e0190635. 10.1371/journal.pone.0190635 29293639 PMC5749833

[B20] Kamiya T. Kai W. Tasumi S. Oka A. Matsunaga T. Mizuno N. (2012). A trans-species missense SNP in Amhr2 is associated with sex determination in the tiger Pufferfish, *Takifugu rubripes* (Fugu). PLoS Genet. 8, e1002798. 10.1371/journal.pgen.1002798 22807687 PMC3395601

[B21] Kitano T. Takamune K. Kobayashi T. Nagahama Y. Abe S. I. (1999). Suppression of P450 aromatase gene expression in sex-reversed males produced by rearing genetic female larvae at a high water temperature during a period of sex differentiation in the Japanese flounder (*Paralichthys olivaceus*). J. Mol. Endocrinol. 23, 167–176. 10.1677/jme.0.0230167 10514554

[B22] Kitano T. Takamune K. Nagahama Y. Abe S. I. (2000). Aromatase inhibitor and 17α-methyltestosterone cause sex-reversal from genetical females to phenotypic males and suppression of P450 aromatase gene expression in Japanese flounder (*Paralichthys olivaceus*). Mol. Reprod. Dev. 56, 1–5. 10.1002/(SICI)1098-2795(200005)56:1<1::AID-MRD1>3.0.CO;2-3 10737961

[B23] Koyama T. Nakamoto M. Morishima K. Yamashita R. Yamashita T. Sasaki K. (2019). A SNP in a steroidogenic enzyme is associated with phenotypic sex in Seriola fishes. Curr. Biol. 29, 1901–1909.e8. e8. 10.1016/j.cub.2019.04.069 31130458

[B24] Li H. Durbin R. (2009). Fast and accurate short read alignment with Burrows-Wheeler Transform. Bioinformatics 5, 1754–1760. 10.1093/bioinformatics/btp324 PMC2705234 19451168

[B25] Li M. Sun Y. Zhao J. Shi H. Zeng S. Ye K. (2015). A tandem duplicate of anti-müllerian hormone with a missense SNP on the Y chromosome is essential for male sex determination in nile Tilapia, *Oreochromis niloticus* . PLoS Genet. 11, e1005678. 10.1371/journal.pgen.1005678 26588702 PMC4654491

[B26] Mankiewicz J. L. Godwin J. Holler B. L. Turner P. M. Murashige R. Shamey R. (2013). Masculinizing effect of background color and cortisol in a flatfish with environmental sex-determination. Integr. Comp. Biol. 53, 755–765. 10.1093/icb/ict093 23946267

[B27] Manly K. F. Olson J. M. (1999). Overview of QTL mapping software and introduction to Map Manager QT. Mamm. Genome 10, 327–334. 10.1007/s003359900997 10087288

[B28] Martínez P. Robledo D. Taboada X. Blanco A. Moser M. Maroso F. (2021). A genome-wide association study, supported by a new chromosome-level genome assembly, suggests *sox2* as a main driver of the undifferentiatiated ZZ/ZW sex determination of turbot (*Scophthalmus maximus*). Genomics 113, 1705–1718. 10.1016/j.ygeno.2021.04.007 33838278

[B29] Miyoshi K. Hattori R. S. Strüssmann C. A. Yokota M. Yamamoto Y. (2020). Phenotypic/genotypic sex mismatches and temperature-dependent sex determination in a wild population of an Old World atherinid, the cobaltcap silverside *Hypoatherina tsurugae* . Mol. Ecol. 29, 2349–2358. 10.1111/mec.15490 32474976

[B30] Pan Q. Feron R. Jouanno E. Darras H. Herpin A. Koop B. (2021). The rise and fall of the ancient northern pike master sex determining gene. Elife 10, e62858–50. 10.7554/eLife.62858 33506762 PMC7870143

[B31] Pan Q. Feron R. Yano A. Guyomard R. Jouanno E. Vigouroux E. (2019). Identification of the master sex determining gene in Northern pike (*Esox lucius*) reveals restricted sex chromosome differentiation. PLoS Genet. 15, e1008013. 10.1371/journal.pgen.100801310.1101/549527 31437150 PMC6726246

[B32] Peterson B. C. David K. B. (2012). Effects of gender and sex hormones on disease susceptibility of channel catfish to *Edwardsiella ictaluri* . J. World Aquac. Soc. 43, 733–738. 10.1111/j.1749-7345.2012.00588.x

[B33] Sakamoto T. Danzmann R. G. Gharbi K. Howard P. Ozaki A. Khoo S. K. (2000). A microsatellite linkage map of rainbow trout (*Oncorhynchus mykiss*) characterized by large sex-specific differences in recombination rates. Genetics 155, 1331–1345. 10.1093/genetics/155.3.1331 10880492 PMC1461176

[B34] Sarida M. Hattori R. S. Zhang Y. Yamamoto Y. Strüssmann C. A. (2019). Spatiotemporal correlations between *amh* and *cyp19a1a* transcript expression and apoptosis during gonadal sex differentiation of pejerrey. Sex. Dev. 13, 99–108. 10.1159/000498997 30913555

[B35] Sawamura R. Osafune N. Murakami T. Furukawa F. Kitano T. (2017). Generation of biallelic F0 mutants in medaka using the CRISPR/Cas9 system. Genes Cells. 22, 756–763. 10.1111/gtc.12511 28707405

[B36] Song W. Xie Y. Sun M. Li X. Fitzpatrick C. K. Vaux F. (2021). A duplicated *amh* is the master sex-determining gene for *Sebastes* rockfish in the Northwest Pacific. Open Biol. 11, 11210063210063. 10.1098/rsob.210063 PMC8277470 34255977

[B37] Takehana Y. Matsuda M. Myosho T. Suster M. L. Kawakami K. Shin-I T. (2014). Co-option of Sox3 as the male-determining factor on the y chromosome in the fish *Oryzias dancena* . Nat. Commun. 5, 4157–4210. 10.1038/ncomms5157 24948391

[B38] Voorrips R. E. (2002). MapChart: Software for the graphical presentation of linkage maps and QTLs. J. Hered. 93, 77–78. 10.1093/jhered/93.1.77 12011185

[B39] Yamaguchi T. Yoshinaga N. Yazawa T. Gen K. Kitano T. (2010). Cortisol is involved in temperature-dependent sex determination in the Japanese flounder. Endocrinology 151, 3900–3908. 10.1210/en.2010-0228 20534725

[B40] Yamamoto E. (1999). Studies on sex-manipulation and production of cloned populations in hirame, *Paralichthys olivaceus* (Temminck et Schlegel). Aquaculture 173, 235–246. 10.1016/s0044-8486(98)00448-7

[B41] Yamamoto E. (1995). Studies on sex-manipulation and production of cloned populations in hirame flounder, *Paralichthys olivaceus* (Temminek et Schlegel). Bull. Tottori Pref. Fish. Exp. Stn. 34, 1–145.

[B42] Yamamoto Y. Zhang Y. Sarida M. Hattori R. S. Strüssmann C. A. (2014). Coexistence of genotypic and temperature-dependent sex determination in pejerrey Odontesthes bonariensis. PLoS One 9, e102574. 10.1371/journal.pone.0102574 25036903 PMC4103838

[B43] Yano A. Nicol B. Jouanno E. Quillet E. Fostier A. Guyomard R. (2013). The sexually dimorphic on the Y-chromosome gene (*sdY*) is a conserved male-specific Y-chromosome sequence in many salmonids. Evol. Appl. 6, 486–496. 10.1111/eva.12032 23745140 PMC3673476

[B44] Yoshinaga N. Shiraishi E. Yamamoto T. Iguchi T. Abe S. I. Kitano T. (2004). Sexually dimorphic expression of a teleost homologue of Müllerian inhibiting substance during gonadal sex differentiation in Japanese flounder, *Paralichthys olivaceus* . Biochem. Biophys. Res. Commun. 322, 508–513. 10.1016/j.bbrc.2004.07.162 15325259

