# Supplementary material for: Y-specific amh allele, amhy, is the master sex-determining gene in Japanese flounder Paralichthys olivaceus
Source: Front Genet. 2022 Sep 16;13:1007548. doi: 10.3389/fgene.2022.1007548 (PMC9523440; doi:10.3389/fgene.2022.1007548)
Supplement: Supplementary file 1 [file Table1.DOCX]

Supplementary Material

# Supplementary Tables

| **Supplemental material** |  |  |  |  |  |  |  |  |
| --- | --- | --- | --- | --- | --- | --- | --- | --- |
| Supplementary Table S1. Total length of *P. olivaceus* larvae used for expression analysis. Values represent the measurement of the most representative size.   \| **Age**  **(dah)** \| **Total length**  **(mm)** \|  \| **Age**  **(dah)** \| **Total length**  **(mm)** \| \| --- \| --- \| --- \| --- \| --- \| \| 20 \| 6.75 \|  \| 55 \| 24.13 \| \| 25 \| 7.8 \|  \| 60 \| 26.51 \| \| 30 \| 11.04 \|  \| 65 \| 30.68 \| \| 35 \| 14.77 \|  \| 70 \| 39.56 \| \| 40 \| 15.7 \|  \| 80 \| 60.67 \| \| 45 \| 17.6 \|  \| 100 \| 76.73 \| \| 50 \| 19.33 \|  \|  \|  \| |  |  |  |  |  |  |  |  |
| Supplementary Table S2. List of primers used for isolation of *amhy* and *amhx* gene sequences.   \| **Purpose** \| **Oligo name** \| **Oligo sequence (5 – 3)** \| **Amplification conditions** \| \| --- \| --- \| --- \| --- \| \| *amhy* 5’ upstream region sequencing \| *amh* JF1 \| GAAACTGTTACATGCGGATCTATC \| 1x 95°C 2 min; 35x 95°C 30 sec, 60°C 30 sec and 72°C 1 min; 1x 72°C 5 min \| \| *amh* RY \| TCCCACCTTCTGAATGCTTGTAG \| \| Sequencing of *amhx* 5’ upstream region \| *amh* JF1 \| GAAACTGTTACATGCGGATCTATC \| 1x 95°C 2 min; 35x 95°C 30 sec, 60°C 30 sec and 72°C 1 min; 1x 72°C 5 min \| \| *amh* RX \| CATCAACCAGGGTTTCAAGATT \| \| Sequencing of *amhy* exons and introns \| *amh* FY-2 \| CTACAAGCATTCAGAAGGTGGGA \| 1x 95°C 2 min; 35x 98°C 10 sec and 68°C 3 min; 1x 68°C 5 min \| \| *amh* BDR6 \| TTAGCTGTCACAGCAGCAGGACT \| \| Sequencing of *amhx* exons and introns \| *amh* FX \| AATCTTGAAACCCTGGTTGATG \| 1x 95°C 2 min; 35x 98°C 10 sec and 68°C 3 min; 1x 68°C 5 min \| \| *amh* BDR6 \| TTAGCTGTCACAGCAGCAGGACT \| \| 5’ RACE \| Amh5UTRRv1 \| CGCAGACCCCAAACAGAGCAAAACT \| Following RACE protocol of manufacturer \| \| Amh5UTRRv2 \| GCACTCCAGTTGGATGCAAAACCTC \| \| 3’ RACE \| *amh* BDF6-2 \| CGAACATGGTACTTATCTCTCCG \| Following RACE protocol of manufacturer \| \| *amh* BDF7 \| CAGACAAATCCTGTGACTGCTC \|   Supplementary Table S3. List of primers and probes used for expression analysis by qRT-PCR.   \| **Gene** \| **Oligo name** \| **Oligo sequence (5 – 3)** \| **Amplification conditions** \| \| --- \| --- \| --- \| --- \| \| *amhy*  (TaqMan) \| Fw: *amh* TF3 \| GCAGCACTGACAGTTTCTCATC \| 1x 95°C 30 sec; 40x 95°C 5 sec and 70°C 1 min \| \| Rv: *amh* TR3 \| CCTCAGTCACAAGCACTCCA \| \| Probe: amhyTY \| CCTTGCACCGTGCTATGTGG \| \| *amhx*  (TaqMan) \| Fw: *amh* TF3 \| GCAGCACTGACAGTTTCTCATC \| 1x 95°C 30 sec; 40x 95°C 5 sec and 70°C 1 min \| \| Rv: *amh* TR3 \| CCTCAGTCACAAGCACTCCA \| \| Probe: amhxCX \| CCTTGCACCGTGCTACGTGG \| \| *amhrII*  (SyBr) \| Fw: amhrIIF \| TCTGCAGTTTGCTGAATATGGTTCT \| 1x 95°C 30 sec; 40x 95°C 5 sec and 60°C 1 min \| \| Rv: amhrIIR \| TGTGAGCCACAGGAGGTTTG \| \| *cyp19a1a* (SyBr) \| Fw: cyp19a1aF \| CGAGAGATTGACACTGTAGTAGGTG \| 1x 95°C 30 sec; 40x 95°C 5 sec and 60°C 1 min \| \| Rv: cyp19a1aR \| GAGGATGATGTTTGTGCCCTTTG \| \| *elf1a*  (SyBr) \| Fw: elf1a-F2 \| TGGTGTGAAGCAGCTCATTG \| 1x 95°C 30 sec; 40x 95°C 5 sec and 60°C 1 min \| \| Rv: elf1a-R \| ATCCAGAGCATCCAGCAGTG \|   Supplementary Table S4. List of 181 predicted genes identified in the contig containing the sex-linked microsatellite markers.   \| \| **ID** \| **Reference** \| **Start** \| **End** \| **Strand** \| **Uniprot code** \| **E-value** \| **Gene Symbol** \| \| --- \| --- \| --- \| --- \| --- \| --- \| --- \| --- \| \| gene1 \| scaffold126 \| 1221 \| 18638 \| - \| P18850 \| 1.23E-69 \| ATF6A \| \| gene2 \| scaffold126 \| 25067 \| 49970 \| + \| Q6DHP9 \| 0 \| RXRGB \| \| gene3 \| scaffold126 \| 52585 \| 61456 \| + \| Q04650 \| 2.44E-55 \| LMX1A \| \| gene4 \| scaffold126 \| 63758 \| 70771 \| - \| Q01102 \| 1.42E-103 \| LYAM3 \| \| gene5 \| scaffold126 \| 71594 \| 72526 \| + \| P35072 \| 2.92E-09 \| TCB1 \| \| gene6 \| scaffold126 \| 74109 \| 81488 \| - \| Q95LG1 \| 1.12E-141 \| LYAM2 \| \| gene7 \| scaffold126 \| 84092 \| 103158 \| + \| Q5TYP4 \| 0 \| CA112 \| \| gene8 \| scaffold126 \| 104456 \| 111106 \| - \| Q8IZE3 \| 1.29E-180 \| PACE1 \| \| gene9 \| scaffold126 \| 111997 \| 126368 \| + \| Q8VI36 \| 5.74E-76 \| PAXI \| \| gene10 \| scaffold126 \| 127823 \| 144292 \| + \| E7F4V6 \| 7.8E-121 \| TIKI2 \| \| gene11 \| scaffold126 \| 162624 \| 191714 \| + \| B1ATG9 \| 1.15E-54 \| TIKI2 \| \| gene12 \| scaffold126 \| 195639 \| 219090 \| + \| E7F4V6 \| 2.27E-45 \| TIKI2 \| \| gene13 \| scaffold126 \| 286613 \| 287716 \| - \| Q90WN4 \| 2.21E-116 \| FOXD2 \| \| gene14 \| scaffold126 \| 295716 \| 298850 \| - \| Q9PTK2 \| 2.96E-147 \| FOXE4 \| \| gene15 \| scaffold126 \| 300036 \| 308014 \| + \| Q9BT25 \| 2.46E-34 \| HAUS8 \| \| gene16 \| scaffold126 \| 308922 \| 360552 \| + \| Q8IZJ3 \| 0 \| CPMD8 \| \| gene17 \| scaffold126 \| 362102 \| 382305 \| + \| O70142 \| 0 \| SHC2 \| \| gene18 \| scaffold126 \| 384988 \| 399373 \| - \| Q80VM7 \| 4.8E-80 \| ANR24 \| \| gene19 \| scaffold126 \| 402192 \| 424208 \| + \| Q7M4L6 \| 5.3E-66 \| SHF \| \| gene20 \| scaffold126 \| 433349 \| 438655 \| - \| Q99500 \| 9.17E-124 \| S1PR3 \| \| gene21 \| scaffold126 \| 440554 \| 460779 \| + \| Q80U19 \| 0 \| DAAM2 \| \| gene22 \| scaffold126 \| 464703 \| 491954 \| - \| Q62415 \| 0 \| ASPP1 \| \| gene23 \| scaffold126 \| 497048 \| 522250 \| - \| Q6DFV7 \| 0 \| NCOA7 \| \| gene24 \| scaffold126 \| 525079 \| 532002 \| + \| Q91677 \| 1.8E-168 \| GATA4 \| \| gene25 \| scaffold126 \| 541190 \| 555126 \| + \| P53798 \| 1.16E-165 \| FDFT \| \| gene26 \| scaffold126 \| 561506 \| 571046 \| + \| Q13237 \| 9.68E-143 \| KFP2 \| \| gene27 \| scaffold126 \| 579869 \| 607119 \| - \| Q920B6 \| 0 \| KCNK2 \| \| gene28 \| scaffold126 \| 613983 \| 624337 \| + \| O95841 \| 1.03E-60 \| ANGL1 \| \| gene29 \| scaffold274 \| 58544 \| 66061 \| + \| Q8N6K7 \| 0.000000121 \| SAMD3 \| \| gene30 \| scaffold274 \| 113301 \| 140150 \| - \| Q09LZ8 \| 2.15E-38 \| CBPC6 \| \| gene31 \| scaffold274 \| 178582 \| 179217 \| - \| Q95SX7 \| 4.01E-09 \| RTBS \| \| gene32 \| scaffold274 \| 181207 \| 196679 \| + \| Q3TL44 \| 2.49E-160 \| NLRX1 \| \| gene33 \| scaffold274 \| 202061 \| 204197 \| - \| Q6P3L0 \| 0.000255 \| MVP \| \| gene34 \| scaffold274 \| 205596 \| 216077 \| + \| Q3TL44 \| 8.44E-44 \| NLRX1 \| \| gene35 \| scaffold274 \| 217750 \| 222411 \| - \| P03934 \| 6.93E-08 \| TC1A \| \| gene36 \| scaffold274 \| 231538 \| 242257 \| + \| Q5RCF7 \| 4.11E-32 \| NHRF3 \| \| gene37 \| scaffold274 \| 243170 \| 243745 \| + \| Q3T0X8 \| 0.000000154 \| NHRF3 \| \| gene38 \| scaffold274 \| 261763 \| 262497 \| + \| Q587J6 \| 5.25E-08 \| LITD1 \| \| gene39 \| scaffold274 \| 268516 \| 269550 \| - \| Q86UP8 \| 2.27E-74 \| GTD2A \| \| gene40 \| scaffold274 \| 280834 \| 299971 \| + \| P11260 \| 0.00000138 \| LORF1 \| \| gene41 \| scaffold274 \| 302791 \| 309085 \| - \| P21328 \| 3.65E-12 \| RTJK \| \| gene42 \| scaffold274 \| 313654 \| 330303 \| + \| Q99NI3 \| 2.74E-22 \| GT2D2 \| \| gene43 \| scaffold274 \| 334179 \| 334459 \| + \| Q04690 \| 0.000000392 \| NF1 \| \| gene44 \| scaffold274 \| 336903 \| 337373 \| - \| Q80WQ9 \| 9.87E-17 \| ZBED4 \| \| gene45 \| scaffold274 \| 360162 \| 365537 \| + \| Q04690 \| 2.83E-43 \| NF1 \| \| gene46 \| scaffold274 \| 382009 \| 390985 \| - \| O96006 \| 2.21E-43 \| ZBED1 \| \| gene47 \| scaffold274 \| 393002 \| 459889 \| + \| P21359 \| 0 \| NF1 \| \| gene48 \| scaffold301 \| 348 \| 5000 \| + \| O57321 \| 0 \| SEAAT1 \| \| gene49 \| scaffold301 \| 7406 \| 20419 \| - \| Q9NR33 \| 5.27E-38 \| DPOE4 \| \| gene50 \| scaffold301 \| 21578 \| 26659 \| + \| Q8BHI9 \| 7.65E-158 \| NIM1 \| \| gene51 \| scaffold301 \| 31734 \| 32655 \| - \| O35903 \| 2.32E-10 \| CCL25 \| \| gene52 \| scaffold301 \| 35015 \| 40710 \| + \| Q2T9Y6 \| 2.17E-113 \| GSH0 \| \| gene53 \| scaffold301 \| 43676 \| 50598 \| + \| Q5QJE6 \| 4.28E-82 \| TDIF2 \| \| gene54 \| scaffold301 \| 55988 \| 64812 \| - \| Q99MU5 \| 6.71E-67 \| SPAT6 \| \| gene55 \| scaffold301 \| 75462 \| 79219 \| - \| Q5VU57 \| 8.11E-47 \| CBPC6 \| \| gene56 \| scaffold301 \| 85640 \| 97917 \| - \| Q09LZ8 \| 1.34E-31 \| CBPC6 \| \| gene57 \| scaffold301 \| 128328 \| 135443 \| - \| Q8C6D4 \| 7.54E-155 \| BEND5 \| \| gene58 \| scaffold301 \| 174466 \| 180274 \| - \| Q3UH53 \| 3.8E-12 \| SDK1 \| \| gene59 \| scaffold301 \| 288301 \| 297013 \| + \| Q6NYK3 \| 5.05E-152 \| SPRE2 \| \| gene60 \| scaffold301 \| 302707 \| 320736 \| - \| Q7SXW6 \| 0 \| ARP2A \| \| gene61 \| scaffold301 \| 323095 \| 324626 \| - \| Q8NF99 \| 6.74E-32 \| ZN397 \| \| gene62 \| scaffold301 \| 330295 \| 361796 \| - \| Q924I2 \| 0 \| M4K3 \| \| gene63 \| scaffold301 \| 369321 \| 381058 \| - \| Q9BTF0 \| 5.08E-28 \| THUM2 \| \| gene64 \| scaffold301 \| 395823 \| 417722 \| - \| P70414 \| 1.89E-23 \| NAC1 \| \| gene65 \| scaffold301 \| 423947 \| 435566 \| - \| P70414 \| 5.45E-11 \| NAC1 \| \| gene66 \| scaffold318 \| 4080 \| 18736 \| - \| Q6DE55 \| 0 \| ARHGAP45 \| \| gene67 \| scaffold318 \| 20719 \| 40834 \| - \| O95049 \| 3.44E-150 \| TJP3 \| \| gene68 \| scaffold318 \| 45072 \| 91466 \| - \| A0JND4 \| 0 \| SBNO2 \| \| gene69 \| scaffold318 \| 108196 \| 133552 \| + \| Q80WM4 \| 2.43E-133 \| HPLN4 \| \| gene70 \| scaffold318 \| 134550 \| 162058 \| - \| Q5IS41 \| 4.11E-56 \| NCAN \| \| gene71 \| scaffold318 \| 228281 \| 249109 \| - \| O14594 \| 1.09E-114 \| NCAN \| \| gene72 \| scaffold318 \| 312834 \| 332405 \| - \| O14593 \| 6.65E-84 \| RFXANK \| \| gene73 \| scaffold318 \| 333004 \| 335122 \| + \| A3KQI3 \| 3.23E-73 \| BORCS8 \| \| gene74 \| scaffold318 \| 336342 \| 339610 \| - \| A6NGB7 \| 8.22E-15 \| TMEM221 \| \| gene75 \| scaffold318 \| 343022 \| 355459 \| + \| Q02080 \| 6.97E-71 \| MEF2B \| \| gene76 \| scaffold318 \| 361704 \| 368173 \| - \| Q0V947 \| 0 \| TMEM161 \| \| gene77 \| scaffold318 \| 404953 \| 425169 \| - \| Q0P483 \| 2.89E-99 \| SLC25A42 \| \| gene78 \| scaffold318 \| 429702 \| 432106 \| - \| O96006 \| 1.08E-57 \| ZBED1 \| \| gene79 \| scaffold318 \| 448069 \| 452583 \| + \| P04323 \| 3.14E-97 \| POL3 \| \| gene80 \| scaffold318 \| 453172 \| 459494 \| + \| Q3T0E8 \| 8.61E-18 \| CALML4 \| \| gene81 \| scaffold318 \| 464344 \| 465699 \| - \| Q8BGE9 \| 7.36E-122 \| RLN3 \| \| gene82 \| scaffold318 \| 498124 \| 501901 \| - \| Q9DCL2 \| 7.54E-75 \| CIAO2A \| \| gene83 \| scaffold318 \| 502331 \| 524230 \| + \| Q3UHA3 \| 0 \| SPG11 \| \| gene84 \| scaffold318 \| 528788 \| 531513 \| + \| P22770 \| 0 \| CHRNA7 \| \| gene85 \| scaffold318 \| 532631 \| 537652 \| + \| Q75HV1 \| 1.26E-85 \| KIN6 \| \| gene86 \| scaffold318 \| 537936 \| 542099 \| - \| Q9NRM2 \| 0 \| ZNF277 \| \| gene87 \| scaffold318 \| 554846 \| 564506 \| + \| Q6AZD4 \| 2.62E-12 \| IMMP2L \| \| gene88 \| scaffold318 \| 593697 \| 595805 \| - \| Q5R482 \| 0 \| NLRR3 \| \| gene89 \| scaffold346 \| 5305 \| 26375 \| - \| O93567 \| 1.22E-160 \| ZBT7A \| \| gene90 \| scaffold346 \| 30202 \| 55581 \| - \| F1R4C4 \| 0 \| PIAS4 \| \| gene91 \| scaffold346 \| 56622 \| 64808 \| - \| F1R4C4 \| 0 \| PIAS4 \| \| gene92 \| scaffold346 \| 67935 \| 81082 \| + \| P32183 \| 5.57E-27 \| FOXA3 \| \| gene93 \| scaffold346 \| 93101 \| 94153 \| + \| O95948 \| 2.42E-123 \| ONEC2 \| \| gene94 \| scaffold346 \| 98348 \| 141898 \| - \| Q148W0 \| 0 \| AT8B1 \| \| gene95 \| scaffold346 \| 144393 \| 145391 \| + \| Q16649 \| 4.44E-20 \| NFIL3 \| \| gene96 \| scaffold346 \| 151087 \| 151776 \| + \| Q68EL6 \| 1.83E-28 \| NFIL3 \| \| gene97 \| scaffold346 \| 162011 \| 163837 \| - \| Q6GQU6 \| 0 \| LIGO3 \| \| gene98 \| scaffold346 \| 225456 \| 226376 \| + \| Q8NCU7 \| 0.0000932 \| C2CD4A \| \| gene99 \| scaffold346 \| 230339 \| 233952 \| + \| Q9H792 \| 3.52E-12 \| PEAK1 \| \| gene100 \| scaffold346 \| 235578 \| 238859 \| - \| Q9YI98 \| 2.3E-92 \| OAZ1 \| \| gene101 \| scaffold346 \| 243918 \| 246118 \| - \| P79295 \| 4.77E-14 \| MIS \| \| gene102 \| scaffold346 \| 255308 \| 274862 \| - \| Q8TEK3 \| 0 \| DOT1L \| \| gene103 \| scaffold346 \| 281773 \| 311967 \| - \| P55199 \| 0 \| ELL \| \| gene104 \| scaffold346 \| 314506 \| 320975 \| - \| Q14318 \| 8.61E-140 \| FKBP8 \| \| gene105 \| scaffold346 \| 323630 \| 354292 \| - \| Q98948 \| 9.25E-38 \| SSBP3 \| \| gene106 \| scaffold346 \| 463524 \| 468631 \| - \| Q0VCT2 \| 1.75E-20 \| TM59L \| \| gene107 \| scaffold346 \| 469951 \| 472824 \| - \| Q9UK28 \| 1.39E-34 \| TM59L \| \| gene108 \| scaffold346 \| 479555 \| 497578 \| + \| O75462 \| 4.21E-168 \| CRLF1 \| \| gene109 \| scaffold346 \| 500061 \| 508332 \| - \| Q6YC49 \| 4.93E-117 \| S35A3 \| \| gene110 \| scaffold346 \| 511640 \| 515191 \| - \| Q04721 \| 0 \| NOTC2 \| \| gene111 \| scaffold346 \| 515968 \| 561610 \| - \| Q04721 \| 0 \| NOTC2 \| \| gene112 \| scaffold346 \| 566678 \| 583762 \| - \| Q7SXP0 \| 1.63E-150 \| S22BB \| \| gene113 \| scaffold346 \| 584662 \| 595809 \| + \| Q6GQB9 \| 0 \| EDEM3 \| \| gene114 \| scaffold346 \| 611725 \| 625275 \| + \| Q8K207 \| 2.62E-10 \| CA021 \| \| gene115 \| scaffold346 \| 630429 \| 636404 \| - \| Q8BVN8 \| 2.43E-76 \| IDLC \| \| gene116 \| scaffold346 \| 640872 \| 695197 \| + \| Q6P112 \| 0 \| RGL1 \| \| gene117 \| scaffold346 \| 701099 \| 710936 \| - \| Q2HJ19 \| 0 \| AB17A \| \| gene118 \| scaffold346 \| 713980 \| 760573 \| - \| O60664 \| 5.86E-93 \| PLIN3 \| \| gene119 \| scaffold362 \| 18546 \| 27556 \| + \| Q9P055 \| 0 \| JKAMP \| \| gene120 \| scaffold362 \| 29913 \| 47345 \| + \| Q03669 \| 3.15E-46 \| AT2A2 \| \| gene121 \| scaffold362 \| 49932 \| 120209 \| - \| O70141 \| 0 \| SEM6B \| \| gene122 \| scaffold362 \| 173104 \| 188939 \| - \| Q4P0H7 \| 0.0000749 \| BBP \| \| gene123 \| scaffold362 \| 190026 \| 195130 \| - \| P14381 \| 1.93E-49 \| YTX2 \| \| gene124 \| scaffold362 \| 196570 \| 228004 \| - \| P14381 \| 2.07E-64 \| YTX2 \| \| gene125 \| scaffold362 \| 260357 \| 262868 \| + \| Q8C4G9 \| 2.5E-141 \| AGRA1 \| \| gene126 \| scaffold362 \| 266175 \| 280324 \| + \| Q5FWU8 \| 6.76E-14 \| ZMY10 \| \| gene127 \| scaffold362 \| 285712 \| 305901 \| + \| Q0KK55 \| 1.03E-59 \| KNDC1 \| \| gene128 \| scaffold362 \| 306444 \| 328381 \| + \| Q76NI1 \| 2.04E-153 \| KNDC1 \| \| gene129 \| scaffold362 \| 330835 \| 331730 \| + \| Q91926 \| 2.23E-30 \| VENT1 \| \| gene130 \| scaffold362 \| 363202 \| 375451 \| - \| O14792 \| 6.41E-129 \| HS3S1 \| \| gene131 \| scaffold410 \| 177634 \| 178616 \| - \| B2RZ86 \| 0.026 \| CCD93 \| \| gene132 \| scaffold410 \| 240447 \| 249728 \| - \| Q6NYU6 \| 6.51E-119 \| MIDN \| \| gene133 \| scaffold410 \| 259837 \| 285591 \| - \| Q504T8 \| 0.000505 \| MIDN \| \| gene134 \| scaffold410 \| 286898 \| 291466 \| - \| Q9D3D9 \| 1.39E-72 \| ATPD \| \| gene135 \| scaffold410 \| 294837 \| 304889 \| + \| Q8N350 \| 3.77E-98 \| CBARP \| \| gene136 \| scaffold410 \| 340756 \| 344210 \| - \| P02316 \| 0.000152 \| p05114 \| \| gene137 \| scaffold410 \| 349607 \| 359814 \| + \| Q58DU7 \| 3.94E-46 \| SH3L1 \| \| gene138 \| scaffold410 \| 367056 \| 382205 \| + \| Q6ZP80 \| 4.25E-55 \| TM182 \| \| gene139 \| scaffold717 \| 488 \| 6323 \| - \| P28173 \| 1.04E-129 \| PUR1 \| \| gene140 \| scaffold717 \| 7049 \| 16875 \| + \| P38024 \| 0 \| PUR6 \| \| gene141 \| scaffold717 \| 19527 \| 23859 \| - \| Q8IYK8 \| 2.17E-49 \| REM2 \| \| gene142 \| scaffold717 \| 26688 \| 33753 \| - \| A7Z017 \| 1.58E-45 \| MAF \| \| gene143 \| scaffold717 \| 39851 \| 68303 \| - \| Q5XHY1 \| 0 \| CARL3 \| \| gene144 \| scaffold717 \| 70224 \| 78461 \| + \| P98160 \| 0.00000205 \| PGBM \| \| gene145 \| scaffold717 \| 80767 \| 87494 \| + \| Q92696 \| 1.36E-143 \| PGTA \| \| gene146 \| scaffold717 \| 93331 \| 113364 \| + \| P22758 \| 3.39E-121 \| TGM1 \| \| gene147 \| scaffold717 \| 119423 \| 123926 \| - \| Q9JI35 \| 6.78E-140 \| HRH3 \| \| gene148 \| scaffold717 \| 127351 \| 136358 \| - \| A9UMG5 \| 4.31E-42 \| IMPCT \| \| gene149 \| scaffold717 \| 138284 \| 167656 \| + \| Q9BXW6 \| 0 \| OSBL1 \| \| gene150 \| scaffold717 \| 169402 \| 188273 \| - \| Q61789 \| 1.46E-126 \| LAMA3 \| \| gene151 \| scaffold717 \| 189732 \| 201892 \| + \| Q0VD48 \| 0 \| VPS4B \| \| gene152 \| scaffold717 \| 204146 \| 207581 \| - \| Q5F3S2 \| 2.5E-93 \| PKHB2 \| \| gene153 \| scaffold717 \| 208637 \| 209982 \| + \| Q9D8Y8 \| 4.59E-88 \| ING5 \| \| gene154 \| scaffold717 \| 211726 \| 212676 \| - \| A9UL78 \| 2.73E-61 \| CEP19 \| \| gene155 \| scaffold717 \| 213706 \| 215134 \| + \| Q8TBF5 \| 4.84E-17 \| PIGX \| \| gene156 \| scaffold717 \| 216568 \| 227461 \| + \| Q8CIN4 \| 0 \| PAK2 \| \| gene157 \| scaffold717 \| 227896 \| 229401 \| - \| Q8CHJ2 \| 2.32E-67 \| AQP12 \| \| gene158 \| scaffold717 \| 230786 \| 241867 \| + \| Q5RDC9 \| 5.24E-151 \| S35D2 \| \| gene159 \| scaffold717 \| 244775 \| 248697 \| - \| Q15392 \| 0 \| DHC24 \| \| gene160 \| scaffold752 \| 745 \| 23957 \| - \| Q02817 \| 0 \| MUC2 \| \| gene161 \| scaffold752 \| 28084 \| 56639 \| + \| P70080 \| 0 \| TPH1 \| \| gene162 \| scaffold752 \| 61290 \| 66184 \| + \| P20303 \| 1.76E-09 \| GTR1 \| \| gene163 \| scaffold752 \| 83000 \| 87258 \| - \| O00370 \| 6.81E-29 \| LORF2 \| \| gene164 \| scaffold752 \| 109313 \| 112360 \| + \| P03359 \| 2.89E-16 \| POL \| \| gene165 \| scaffold752 \| 126582 \| 138740 \| - \| Q9QY81 \| 2.58E-51 \| PO210 \| \| gene166 \| scaffold752 \| 159495 \| 195184 \| + \| O75553 \| 3.88E-62 \| DAB1 \| \| gene167 \| scaffold752 \| 196647 \| 206953 \| + \| D3ZIE4 \| 5.81E-10 \| FYB1 \| \| gene168 \| scaffold752 \| 209017 \| 221406 \| - \| Q09137 \| 0 \| AAPK2 \| \| gene169 \| scaffold752 \| 222118 \| 251575 \| + \| Q99JY8 \| 1.16E-139 \| PLPP3 \| \| gene170 \| scaffold801 \| 3646 \| 15479 \| - \| O88302 \| 1.9E-143 \| GNA15 \| \| gene171 \| scaffold801 \| 20883 \| 46569 \| + \| P38410 \| 1.38E-89 \| GNAQ \| \| gene172 \| scaffold801 \| 49055 \| 78135 \| + \| P82471 \| 2.78E-109 \| GNAQ \| \| gene173 \| scaffold801 \| 81681 \| 99496 \| + \| Q08117 \| 5.24E-56 \| TLE5 \| \| gene174 \| scaffold801 \| 106018 \| 162292 \| + \| Q9WVB2 \| 7.96E-22 \| TLE2 \| \| gene175 \| scaffold801 \| 176966 \| 206491 \| + \| O42469 \| 0 \| TLE1 \| \| gene176 \| scaffold801 \| 212642 \| 245399 \| - \| O42469 \| 0 \| TLE1 \| \| gene177 \| scaffold970 \| 3953 \| 20632 \| + \| Q90460 \| 0.000000472 \| C166A \| \| gene178 \| scaffold970 \| 33625 \| 64333 \| + \| O65454 \| 0.023 \| FB334 \| \| gene179 \| scaffold970 \| 70631 \| 83196 \| + \| Q7LHG5 \| 0.000000081 \| TY3BI \| \| gene180 \| scaffold970 \| 89080 \| 91295 \| + \| O96006 \| 2.29E-28 \| ZBED1 \| \| gene181 \| scaffold970 \| 201310 \| 202599 \| + \| Q8AVU4 \| 3.05E-147 \| HES1A \| \| \| --- \| --- \| --- \| --- \| --- \| --- \| --- \| --- \| --- \| --- \| --- \| --- \| --- \| --- \| --- \| --- \| --- \| --- \| --- \| --- \| --- \| --- \| --- \| --- \| --- \| --- \| --- \| --- \| --- \| --- \| --- \| --- \| --- \| --- \| --- \| --- \| --- \| --- \| --- \| --- \| --- \| --- \| --- \| --- \| --- \| --- \| --- \| --- \| --- \| --- \| --- \| --- \| --- \| --- \| --- \| --- \| --- \| --- \| --- \| --- \| --- \| --- \| --- \| --- \| --- \| --- \| --- \| --- \| --- \| --- \| --- \| --- \| --- \| --- \| --- \| --- \| --- \| --- \| --- \| --- \| --- \| --- \| --- \| --- \| --- \| --- \| --- \| --- \| --- \| --- \| --- \| --- \| --- \| --- \| --- \| --- \| --- \| --- \| --- \| --- \| --- \| --- \| --- \| --- \| --- \| --- \| --- \| --- \| --- \| --- \| --- \| --- \| --- \| --- \| --- \| --- \| --- \| --- \| --- \| --- \| --- \| --- \| --- \| --- \| --- \| --- \| --- \| --- \| --- \| --- \| --- \| --- \| --- \| --- \| --- \| --- \| --- \| --- \| --- \| --- \| --- \| --- \| --- \| --- \| --- \| --- \| --- \| --- \| --- \| --- \| --- \| --- \| --- \| --- \| --- \| --- \| --- \| --- \| --- \| --- \| --- \| --- \| --- \| --- \| --- \| --- \| --- \| --- \| --- \| --- \| --- \| --- \| --- \| --- \| --- \| --- \| --- \| --- \| --- \| --- \| --- \| --- \| --- \| --- \| --- \| --- \| --- \| --- \| --- \| --- \| --- \| --- \| --- \| --- \| --- \| --- \| --- \| --- \| --- \| --- \| --- \| --- \| --- \| --- \| --- \| --- \| --- \| --- \| --- \| --- \| --- \| --- \| --- \| --- \| --- \| --- \| --- \| --- \| --- \| --- \| --- \| --- \| --- \| --- \| --- \| --- \| --- \| --- \| --- \| --- \| --- \| --- \| --- \| --- \| --- \| --- \| --- \| --- \| --- \| --- \| --- \| --- \| --- \| --- \| --- \| --- \| --- \| --- \| --- \| --- \| --- \| --- \| --- \| --- \| --- \| --- \| --- \| --- \| --- \| --- \| --- \| --- \| --- \| --- \| --- \| --- \| --- \| --- \| --- \| --- \| --- \| --- \| --- \| --- \| --- \| --- \| --- \| --- \| --- \| --- \| --- \| --- \| --- \| --- \| --- \| --- \| --- \| --- \| --- \| --- \| --- \| --- \| --- \| --- \| --- \| --- \| --- \| --- \| --- \| --- \| --- \| --- \| --- \| --- \| --- \| --- \| --- \| --- \| --- \| --- \| --- \| --- \| --- \| --- \| --- \| --- \| --- \| --- \| --- \| --- \| --- \| --- \| --- \| --- \| --- \| --- \| --- \| --- \| --- \| --- \| --- \| --- \| --- \| --- \| --- \| --- \| --- \| --- \| --- \| --- \| --- \| --- \| --- \| --- \| --- \| --- \| --- \| --- \| --- \| --- \| --- \| --- \| --- \| --- \| --- \| --- \| --- \| --- \| --- \| --- \| --- \| --- \| --- \| --- \| --- \| --- \| --- \| --- \| --- \| --- \| --- \| --- \| --- \| --- \| --- \| --- \| --- \| --- \| --- \| --- \| --- \| --- \| --- \| --- \| --- \| --- \| --- \| --- \| --- \| --- \| --- \| --- \| --- \| --- \| --- \| --- \| --- \| --- \| --- \| --- \| --- \| --- \| --- \| --- \| --- \| --- \| --- \| --- \| --- \| --- \| --- \| --- \| --- \| --- \| --- \| --- \| --- \| --- \| --- \| --- \| --- \| --- \| --- \| --- \| --- \| --- \| --- \| --- \| --- \| --- \| --- \| --- \| --- \| --- \| --- \| --- \| --- \| --- \| --- \| --- \| --- \| --- \| --- \| --- \| --- \| --- \| --- \| --- \| --- \| --- \| --- \| --- \| --- \| --- \| --- \| --- \| --- \| --- \| --- \| --- \| --- \| --- \| --- \| --- \| --- \| --- \| --- \| --- \| --- \| --- \| --- \| --- \| --- \| --- \| --- \| --- \| --- \| --- \| --- \| --- \| --- \| --- \| --- \| --- \| --- \| --- \| --- \| --- \| --- \| --- \| --- \| --- \| --- \| --- \| --- \| --- \| --- \| --- \| --- \| --- \| --- \| --- \| --- \| --- \| --- \| --- \| --- \| --- \| --- \| --- \| --- \| --- \| --- \| --- \| --- \| --- \| --- \| --- \| --- \| --- \| --- \| --- \| --- \| --- \| --- \| --- \| --- \| --- \| --- \| --- \| --- \| --- \| --- \| --- \| --- \| --- \| --- \| --- \| --- \| --- \| --- \| --- \| --- \| --- \| --- \| --- \| --- \| --- \| --- \| --- \| --- \| --- \| --- \| --- \| --- \| --- \| --- \| --- \| --- \| --- \| --- \| --- \| --- \| --- \| --- \| --- \| --- \| --- \| --- \| --- \| --- \| --- \| --- \| --- \| --- \| --- \| --- \| --- \| --- \| --- \| --- \| --- \| --- \| --- \| --- \| --- \| --- \| --- \| --- \| --- \| --- \| --- \| --- \| --- \| --- \| --- \| --- \| --- \| --- \| --- \| --- \| --- \| --- \| --- \| --- \| --- \| --- \| --- \| --- \| --- \| --- \| --- \| --- \| --- \| --- \| --- \| --- \| --- \| --- \| --- \| --- \| --- \| --- \| --- \| --- \| --- \| --- \| --- \| --- \| --- \| --- \| --- \| --- \| --- \| --- \| --- \| --- \| --- \| --- \| --- \| --- \| --- \| --- \| --- \| --- \| --- \| --- \| --- \| --- \| --- \| --- \| --- \| --- \| --- \| --- \| --- \| --- \| --- \| --- \| --- \| --- \| --- \| --- \| --- \| --- \| --- \| --- \| --- \| --- \| --- \| --- \| --- \| --- \| --- \| --- \| --- \| --- \| --- \| --- \| --- \| --- \| --- \| --- \| --- \| --- \| --- \| --- \| --- \| --- \| --- \| --- \| --- \| --- \| --- \| --- \| --- \| --- \| --- \| --- \| --- \| --- \| --- \| --- \| --- \| --- \| --- \| --- \| --- \| --- \| --- \| --- \| --- \| --- \| --- \| --- \| --- \| --- \| --- \| --- \| --- \| --- \| --- \| --- \| --- \| --- \| --- \| --- \| --- \| --- \| --- \| --- \| --- \| --- \| --- \| --- \| --- \| --- \| --- \| --- \| --- \| --- \| --- \| --- \| --- \| --- \| --- \| --- \| --- \| --- \| --- \| --- \| --- \| --- \| --- \| --- \| --- \| --- \| --- \| --- \| --- \| --- \| --- \| --- \| --- \| --- \| --- \| --- \| --- \| --- \| --- \| --- \| --- \| --- \| --- \| --- \| --- \| --- \| --- \| --- \| --- \| --- \| --- \| --- \| --- \| --- \| --- \| --- \| --- \| --- \| --- \| --- \| --- \| --- \| --- \| --- \| --- \| --- \| --- \| --- \| --- \| --- \| --- \| --- \| --- \| --- \| --- \| --- \| --- \| --- \| --- \| --- \| --- \| --- \| --- \| --- \| --- \| --- \| --- \| --- \| --- \| --- \| --- \| --- \| --- \| --- \| --- \| --- \| --- \| --- \| --- \| --- \| --- \| --- \| --- \| --- \| --- \| --- \| --- \| --- \| --- \| --- \| --- \| --- \| --- \| --- \| --- \| --- \| --- \| --- \| --- \| --- \| --- \| --- \| --- \| --- \| --- \| --- \| --- \| --- \| --- \| --- \| --- \| --- \| --- \| --- \| --- \| --- \| --- \| --- \| --- \| --- \| --- \| --- \| --- \| --- \| --- \| --- \| --- \| --- \| --- \| --- \| --- \| --- \| --- \| --- \| --- \| --- \| --- \| --- \| --- \| --- \| --- \| --- \| --- \| --- \| --- \| --- \| --- \| --- \| --- \| --- \| --- \| --- \| --- \| --- \| --- \| --- \| --- \| --- \| --- \| --- \| --- \| --- \| --- \| --- \| --- \| --- \| --- \| --- \| --- \| --- \| --- \| --- \| --- \| --- \| --- \| --- \| --- \| --- \| --- \| --- \| --- \| --- \| --- \| --- \| --- \| --- \| --- \| --- \| --- \| --- \| --- \| --- \| --- \| --- \| --- \| --- \| --- \| --- \| --- \| --- \| --- \| --- \| --- \| --- \| --- \| --- \| --- \| --- \| --- \| --- \| --- \| --- \| --- \| --- \| --- \| --- \| --- \| --- \| --- \| --- \| --- \| --- \| --- \| --- \| --- \| --- \| --- \| --- \| --- \| --- \| --- \| --- \| --- \| --- \| --- \| --- \| --- \| --- \| --- \| --- \| --- \| --- \| --- \| --- \| --- \| --- \| --- \| --- \| --- \| --- \| --- \| --- \| --- \| --- \| --- \| --- \| --- \| --- \| --- \| --- \| --- \| --- \| --- \| --- \| --- \| --- \| --- \| --- \| --- \| --- \| --- \| --- \| --- \| --- \| --- \| --- \| --- \| --- \| --- \| --- \| --- \| --- \| --- \| --- \| --- \| --- \| --- \| --- \| --- \| --- \| --- \| --- \| --- \| --- \| --- \| --- \| --- \| --- \| --- \| --- \| --- \| --- \| --- \| --- \| --- \| --- \| --- \| --- \| --- \| --- \| --- \| --- \| --- \| --- \| --- \| --- \| --- \| --- \| --- \| --- \| --- \| --- \| --- \| --- \| --- \| --- \| --- \| --- \| --- \| --- \| --- \| --- \| --- \| --- \| --- \| --- \| --- \| --- \| --- \| --- \| --- \| --- \| --- \| --- \| --- \| --- \| --- \| --- \| --- \| --- \| --- \| --- \| --- \| --- \| --- \| --- \| --- \| --- \| --- \| --- \| --- \| --- \| --- \| --- \| --- \| --- \| --- \| --- \| --- \| --- \| --- \| --- \| --- \| --- \| --- \| --- \| --- \| --- \| --- \| --- \| --- \| --- \| --- \| --- \| --- \| --- \| --- \| --- \| --- \| --- \| --- \| --- \| --- \| --- \| --- \| --- \| --- \| --- \| --- \| --- \| --- \| --- \| --- \| --- \| --- \| --- \| --- \| --- \| --- \| --- \| --- \| --- \| --- \| --- \| --- \| --- \| --- \| --- \| --- \| --- \| --- \| --- \| --- \| --- \| --- \| --- \| --- \| --- \| --- \| --- \| --- \| --- \| --- \| --- \| --- \| --- \| --- \| --- \| --- \| --- \| --- \| --- \| --- \| --- \| --- \| --- \| --- \| --- \| --- \| --- \| --- \| --- \| --- \| --- \| --- \| --- \| --- \| --- \| --- \| --- \| --- \| --- \| --- \| --- \| --- \| --- \| --- \| --- \| --- \| --- \| --- \| --- \| --- \| --- \| --- \| --- \| --- \| --- \| --- \| --- \| --- \| --- \| --- \| --- \| --- \| --- \| --- \| --- \| --- \| --- \| --- \| --- \| --- \| --- \| --- \| --- \| --- \| --- \| --- \| --- \| --- \| --- \| --- \| --- \| --- \| --- \| --- \| --- \| --- \| --- \| --- \| --- \| --- \| --- \| --- \| --- \| --- \| --- \| --- \| --- \| --- \| --- \| --- \| --- \| --- \| --- \| --- \| --- \| --- \| --- \| --- \| --- \| --- \| --- \| --- \| --- \| --- \| --- \| --- \| --- \| --- \| --- \| --- \| --- \| --- \| --- \| --- \| --- \| --- \| --- \| --- \| --- \| --- \| --- \| --- \| --- \| --- \| --- \| --- \| --- \| --- \| --- \| --- \| --- \| --- \| --- \| --- \| --- \| --- \| --- \| --- \| --- \| --- \| --- \| --- \| --- \| --- \| --- \| --- \| --- \| --- \| --- \| --- \| --- \| --- \| --- \| --- \| --- \| --- \| --- \| --- \| --- \| --- \| --- \| --- \| --- \| --- \| --- \| --- \| --- \| --- \| --- \| --- \| --- \| --- \| --- \| --- \| --- \| --- \| --- \| --- \| --- \| --- \| --- \| --- \| --- \| --- \| --- \| --- \| --- \| --- \| --- \| --- \| --- \| --- \| --- \| --- \| --- \| --- \| --- \| --- \| --- \| --- \| --- \| --- \| --- \| --- \| --- \| --- \| --- \| --- \| --- \| --- \| --- \| --- \| --- \| --- \| --- \| --- \| --- \| --- \| --- \| --- \| --- \| --- \| --- \| --- \| --- \| --- \| --- \| --- \| --- \| --- \| --- \| --- \| --- \| --- \| --- \| --- \| --- \| --- \| --- \| --- \| --- \| --- \| --- \| --- \| --- \| --- \| --- \| --- \| --- \| --- \| --- \| --- \| --- \| --- \| --- \| --- \| --- \| --- \| --- \| --- \| --- \| --- \| --- \| --- \| --- \| --- \| --- \| --- \| --- \| --- \| --- \| --- \| --- \| |  |  |  |  |  |  |  |  |
|  |  |  |  |  |  |  |  |  |
|  |  |  |  |  |  |  |  |  |
|  |  |  |  |  |  |  |  |  |
|  |  |  |  |  |  |  |  |  |
|  |  |  |  |  |  |  |  |  |
|  |  |  |  |  |  |  |  |  |
|  |  |  |  |  |  |  |  |  |
|  |  |  |  |  |  |  |  |  |
|  |  |  |  |  |  |  |  |  |
|  |  |  |  |  |  |  |  |  |
|  |  |  |  |  |  |  |  |  |
